# *COMT*-Polymorphisms Modulated Functional Profile of the Fusiform Face Area Contributes to Face-Specific Recognition Ability

**DOI:** 10.1038/s41598-020-58747-4

**Published:** 2020-02-07

**Authors:** Chao Wu, Zonglei Zhen, Lijie Huang, Taicheng Huang, Jia Liu

**Affiliations:** 1 School of Nursing, Peking University Health Science Centre, Beijing, 100191 China; 2 Beijing Key Laboratory of Applied Experimental Psychology, National Demonstration Center for Experimental Psychology Education, Faculty of Psychology, Beijing Normal University, Beijing, 100875 China; 3 State Key Laboratory of Cognitive Neuroscience and Learning and IDG/McGovern Institute for Brain Research, Beijing Normal University, Beijing, 100875 China

**Keywords:** Perception, Human behaviour

## Abstract

Previous studies have shown that face-specific recognition ability (FRA) is heritable; however, the neural basis of this heritability is unclear. Candidate gene studies have suggested that the catechol-O-methyltransferase (COMT) rs4680 polymorphism is related to face perception. Here, using a partial least squares (PLS) method, we examined the multivariate association between 12 genotypes of 4 COMT polymorphisms (rs6269-rs4633-rs4818-rs4680) and multimodal MRI phenotypes in the human fusiform face area (FFA), which selectively responds to face stimuli, in 338 Han Chinese adults (mean age 20.45 years; 135 males). The MRI phenotypes included gray matter volume (GMV), resting-state fractional amplitude of low-frequency fluctuations (fALFF), and face-selective blood-oxygen-level-dependent (BOLD) responses (FS). We found that the first COMT-variant component (PLS1) was positively associated with the FS but negatively associated with the fALFF in the FFA. Moreover, participants with the *COMT* heterozygous-HEA-haplotype showed higher PLS1 FFA-MRI scores, which were positively associated with the FRA in an old/new face recognition task, than those with the *COMT* homozygous HEA haplotype and HEA non-carriers, suggesting that individuals with an appropriate (intermediate) level of dopamine activity in the FFA might have better FRA. In summary, our study provides empirical evidence for the genetic and neural basis for the heritability of face recognition and informs the formation of neural module functional specificity.

## Introduction

Face recognition is a highly developed skill in humans that shows considerable variability between individuals^1–5^. Previous behavioral genetic studies have shown that face recognition ability is heritable^4,6^; however, the neural basis for this heritability is unclear. One candidate cortical region underlying inter-individual variability in face recognition is the human fusiform face area (FFA) especially in the right hemisphere, which shows more significant responses to visual face stimuli than to other non-face stimuli^7^ and represents a critical hub in the face network^8–10^. More importantly, features of the FFA are associated with face-specific recognition ability (FRA)^2,3^. However, information about the effects of genetic variability on FFA structure and function and their relationship with face recognition ability remain limited.

Previous twin studies have demonstrated the higher intra-pair similarity of cortical structure^11^ and functional magnetic resonance imaging (fMRI) responses^11,12^ in the occipitotemporal cortex encompassing the FFA in monozygotic twins than dizygotic twins. Additionally, neural responses to facial expressions are modulated by common genetic variance in a set of regions constituting the face network that include the FFA^13^. These studies suggest that the FFA may be modulated by genetic variations. However, previous studies have not specifically localized the FFA, and it is unclear whether the observed genetic effects are derived from changes in the FFA or other regions. Notably, a previous genome-wide association neuroimaging study explored specific genes contributing to the FFA but failed to identify any significant genome-level correlations between single nucleotide polymorphisms (SNPs) and FFA-responses to facial expressions^14^. One possible reason for this negative result is insufficient statistic power due to a relatively small number of participants (N < 300).

Candidate gene studies have demonstrated the association of the catechol-O-methyltransferase (COMT) gene, located on chromosome 22q11.2, with face recognition^15–19^. The *COMT* gene codes for the COMT enzyme, a protein that degrades catecholamine neurotransmitters and modulates dopamine metabolisms^20^. Among the COMT polymorphisms, Val158Met (rs4680) has been most reported to play a role in face processing^15–24^. For example, individuals with the Met/Met (rs4680) genotype are more sensitive to configural changes in faces than those with other genotypes^15^. Healthy homozygous subjects with the Met allele of rs4680 are better at recognizing facial emotions^16^ and show a stronger bias to perceive neutral faces as expressions of anger^21^ than those with other genotypes. Previous studies also investigated the impact of the COMT gene on the brain activity during face emotional processing^22–25^ and demonstrated that the COMT Met allele seems to contribute to neural substrates of negativity biases^22–24^, and the COMT Val allele is associated with increased amygdala activity in response to fearful/angry facial expressions^25^. It should be noted that several meta-analyses suggest that the effects of COMT Val158Met on cognitive abilities are either very small or nil due to insufficient statistical power, unknown moderators including genetic variations at different loci, publication bias, or effect heterogeneity^26–28^. Therefore, individual studies should pay more attention to the methodological rigor and scientific nature. Accumulating studies have revealed that there are multi-to-multi relationships between genotypes and brain phenotypes linked to learning and cognitive ability^29–31^. Previous studies have indicated that four COMT polymorphisms (rs6269, rs4633, rs4818, and rs4680) have a strong linkage disequilibrium and always function as haplotype blocks to affect dopamine (DA) degrading enzyme activity^32,33^. However, little is known about whether certain patterns of the 4 COMT polymorphisms exert significant effects on the FFA and therefore affect the face recognition ability.

In this study, we employed a combined genetic and neuroimaging approach and incorporated three strategies to improve statistical power without dramatically increasing the number of participants. First, previous studies focused on a single measure of the FFA (i.e., either gray matter volume (GMV) or blood oxygen level-dependent [BOLD] responses); notably, multiple modals and joint modeling can improve sensitivity for identifying disease-associated biological pathways in common complex disorders^34–37^; accordingly, this study used multimodal MRI measures for the FFA (multimodal FFA-MRI data), including GMV, fractional amplitude of low-frequency fluctuations (fALFF) from resting-state fMRI, and BOLD responses specific to faces (face selectivity, FS) from task-state fMRI. Second, the partial least squares (PLS) approach was applied to examine relationships between sets of multivariate measures. This approach has demonstrated utility in studies with relative small samples and problems with multicollinearity^37–40^. Third, bilateral FFAs are involved in face processing simultaneously^5,7,8^, notwithstanding some hemispheric lateralization in their size and functional recruitments^41,42^. We conducted analyses for the right FFA (rFFA) and the left FFA (lFFA) separately to validate the genetic variants that modulate the FFA. Using these three strategies, we identified profiles of 4 COMT polymorphisms that would be robustly associated with profiles of structural or functional FFA, in a population of Han Chinese individuals.

## Results

### Multivariate association between COMT polymorphisms and FFA phenotypes

**Figure 1:**
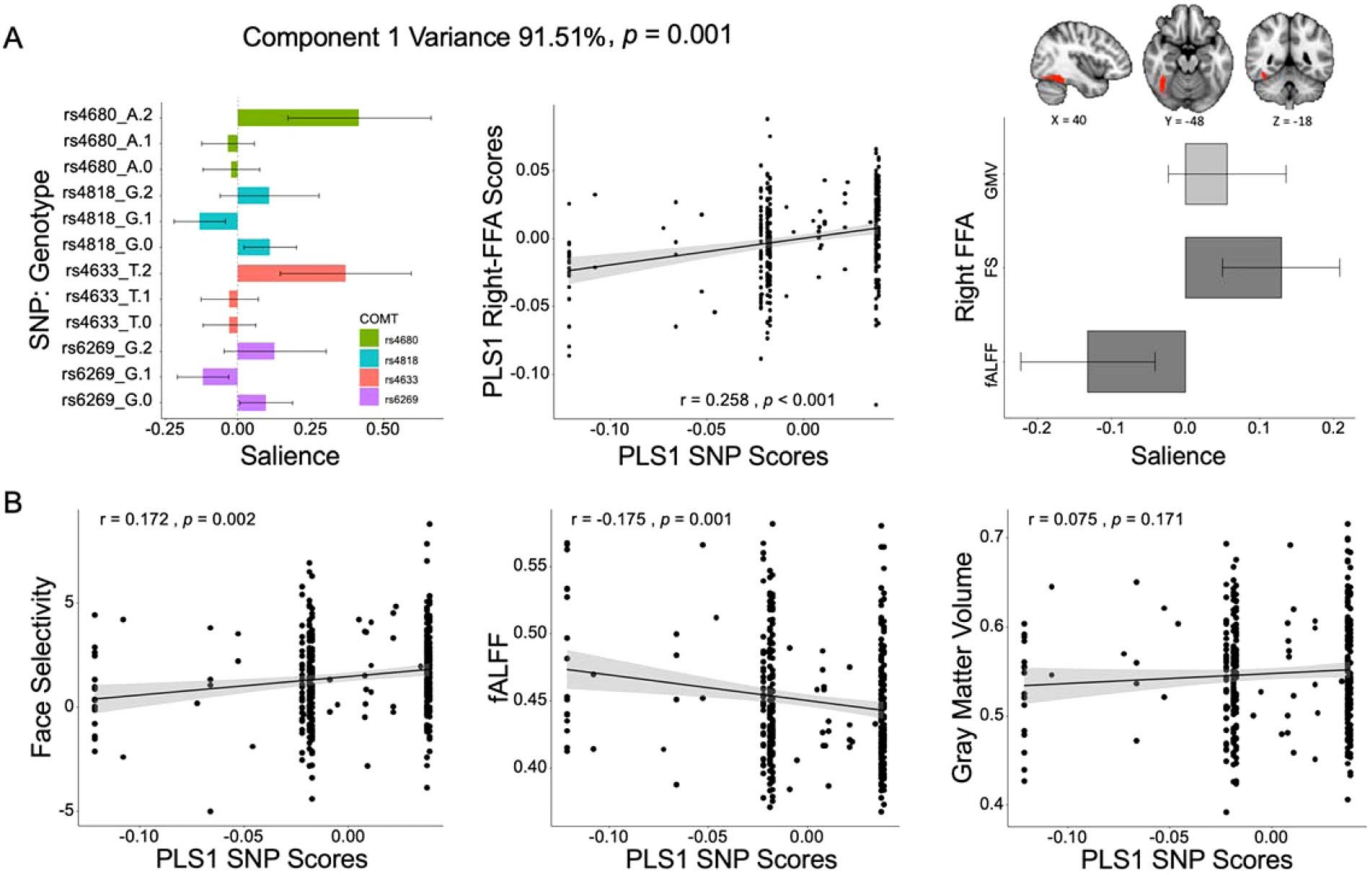
Partial least squares correspondence analysis (PLSCA) between right FFA-MRI (rFFA-MRI) phenotypes and COMT polymorphisms. (**A**) The first pair of latent variables (LVs) from the PLSCA. *Left:* weighted saliences and 95% confidence interval for each COMT genotype; *right*: weighted saliences (weights) and 95% confidence interval for each modal of the rFFA. The rFFA (cluster in red) was defined as a set of contiguous voxels (233 voxels) that were significantly activated in response to faces vs. objects (Z > 2.58, one-tailed, *p* < 0.005, uncorrected) in the lateral mid- and posterior fusiform gyrus in the right hemisphere. **(B**) The first partial least squares component (PLS1) identified a profile (linear combination) of *COMT* genotypes that were positively correlated with face selectivity (FS), negatively correlated with fALFF, and not correlated with GMV of the rFFA.

A PLS correspondence analysis (PLSCA) was conducted to examine whether the 12 *COMT* genotypes contributed to the 3-modal right FFA-MRI phenotype in 338 participants. The PLSCA yielded 3 sets of paired latent variables (LVs) capturing the rFFA-SNP association, ordered by the size of the explained variance (91.51%, 7.0%, and 1.49%). Each component (LV pair) was a linear combination of the weighted COMT SNP scores that most strongly correlated with weighted MRI scores and was orthogonal to each other. The omnibus p value of the model was 0.004 and only the top PLS component (PLS1) was significant (permutation test, *p* = 0.001). Thus, the PLS1 represented a significant association (r = 0.258, *p* < 0.001) between a specifically patterned COMT genotypic profile and a specifically patterned rFFA phenotype (middle panel of Fig. 1A). The power analysis^43^ for the PLS model (Supplementary Table S2) indicated a power of 0.713 for the path between the rFFA and the COMT polymorphisms, which means that we have the probability of 71.3% to detect a significant association between the rFFA and the COMT polymorphisms, given that the association is really there. The component reliability test showed that the rs4680 AA (Met) genotype (salience = 0.417, BSR = 3.33, FDR corrected *p = *0.007, 95%CI [0.17, 0.72]), rs4818 AA genotype (salience = 0.110, BSR = 2.40, FDR corrected *p = *0.040, 95%CI [0.02, 0.20]), rs4818 AG genotype (salience = −0.132, BSR = −2.94, FDR corrected *p = *0.013, 95%CI [−0.22, −0.04]), rs4633 TT genotype (salience = 0.370, BSR = 3.23, FDR corrected *p = *0.007, 95%CI [0.15,0.59]), and rs6269 AG genotype (salience = −0.121, BSR = −2.66, FDR corrected *p = *0.024, 95%CI [−0.21, −0.03]) reliably contributed to the PLS1 COMT-SNP component (left panel of Fig. 1A); the FS (salience = 0.129, BSR = 3.21, FDR corrected *p = *0.007, 95%CI [0.05,0.21]) and fALFF (salience = −0.132, BSR = −2.84, FDR corrected *p = *0.004, 95%CI [−0.22, −0.04]) reliably contributed to the PLS1 rFFA-MRI component (right panel of Fig. 1A). Moreover, the genetic regulation of the rFFA presented a pattern that rs4680 and rs4633 exhibited linear trend of modulation (from recessive homozygote to heterozygote and to dominant homozygote) on the PLS1 rFFA-MRI profile, whereas rs6269 and rs4818 showed a nonlinear relationship with the PLS1 rFFA-MRI profile.

To explain how this profile (linear combination) of the 12 COMT genotypes (i.e., PLS1 SNP profile) modulates the 3 raw MRI measures of the rFFA, we correlated the PLS1 COMT scores with FS, GMV, and fALFF of the rFFA. The results showed that the PLS1 COMT profile was positively correlated with FS (r = 0.172, *p* = 0.002, 95%CI = [0.07, 0.27]), negatively correlated with fALFF (r = −0.175, *p* = 0.001, 95%CI = [−0.28, −0.07]), and not correlated with GMV of the rFFA (r = 0.075, *p* = 0.171, 95%CI = [−0.03, 0.18]) (Fig. 1B). Thus, the *COMT* polymorphism profile seems to be more likely to regulate the functional rather than structural profile of the right FFA.

To valid the results and improve reliability, we conducted the following control analyses. First, we randomly split the voxels within the rFFA into two halves and entered each half of the rFFA measures along with COMT polymorphisms into a PLSCA. We found that both of the rFFA-COMT associations were significant (Supplementary Fig. S1) and the difference between the two associations was not significant (z = 0.227, *p* = 0.821). Moreover, the intra-class correlations (ICC) for the two half GMV, fALFF, FS, PLS1-SNP scores, and PLS1-rFFA scores were 0.985 (95%CI [0.981, 0.988]), 0.995 (95%CI [0.994, 0.996]), 0.989 (95%CI [0.986, 0.991]), 0.999 (95%CI [0.999, 1]), and 0.994 (95%CI [0.993, 0.996]), respectively. Thus, both the rFFA measures and the association pattern between the rFFA phenotypes and the COMT polymorphisms exhibit high reliability. Second, to test whether the threshold for identifying the rFFA affects the main results, we conducted control analyses with 4 different thresholds (z = 2.71, one-tailed uncorrected p < 0.003, FDR corrected p < 0.05; z = 2.58, one-tailed uncorrected p < 0.005; z = 2.34, one-tailed uncorrected p < 0.01; z = 1.96, one-tailed uncorrected p < 0.05) and found that the association between the 12 COMT genotypes and the rFFA-MRI phenotypes was significant in all of the 4 threshold conditions (Fig. S2 in Supplementary Material), suggesting that the threshold for defining the rFFA has little effect on the COMT-rFFA association. Third, to test whether total brain volume affects the current results, we conducted a control PLS analysis after regressing the total brain volume out of the rFFA and found the main results remained (Supplementary Fig. S3). Finally, to test whether the rFFA-COMT association can be reproduced for the left FFA (lFFA), we conducted a PLSCA to explore the association between the 12 COMT genotypes and the 3-modal lFFA-MRI phenotypes. The analysis yielded 3 sets of LVs capturing the lFFA-SNP association, ordered by the size of the explained variance (81.93%, 17.31%, and 0.07%). The covariation between the PLS1 COMT profile and the PLS1 lFFA-MRI profile was marginally significant (permutation test, p = 0.053; upper panel of Supplementary Fig. S4). The ICC for the PLS1 lFFA-SNP scores and PLS1 rFFA-SNP scores was 0.998 (95%CI [0.997, 0.998]), and the ICC for the PLS1 lFFA-MRI scores and rFFA-MRI scores was 0.787 (95%CI [0.736, 0.828]). Moreover, the difference between the lFFA-COMT association and the rFFA-COMT association was not significant (z = 1.331, *p* = 0.183). Thus, the lFFA exhibits a similar association pattern with the COMT polymorphisms as the rFFA does.

### Haplotype analysis

**Figure 2:**
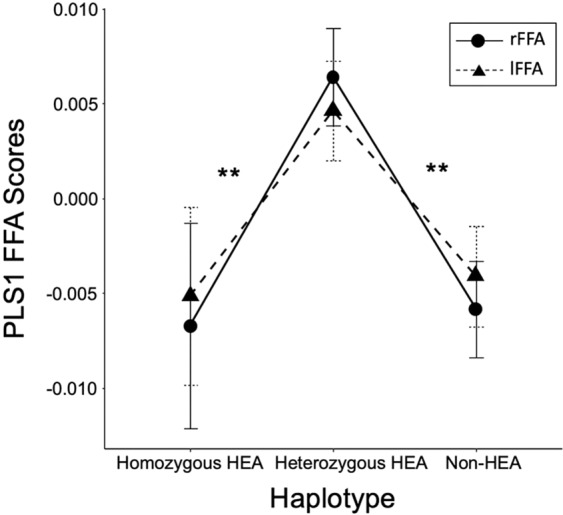
Comparisons of the group-mean PLS1 rFFA-MRI and PLS1 lFFA-MRI scores among three COMT haplotype groups. The difference in the PLS1 FFA-MRI scores was not significant between the hemispheres. Participants with the heterozygous-HEA *COMT* haplotype (intermediate dopamine availability) showed increased face selectivity (FS) and decreased fALFF (positive PLS1 FFA-MRI scores), whereas participants with the COMT homozygous HEA haplotype (high DA degrading enzymatic activity: a low level of DA) and HEA non-carriers (low DA degrading enzymatic activity: a high level of DA) showed decreased FS and increased rFFA-fALFF (negative FFA scores of PLS1). Error bars indicate the standard errors of the mean. ^*^p < 0.05, ^**^p < 0.01 (two-tailed).

Because the four COMT SNPs has been previously found to be in strong linkage disequilibrium and define three common haplotypes with functional consequences on dopamine (DA) degrading enzyme function^32,33^, we performed a haplotype analysis on the 4 genotyped COMT SNPs to inform their functions as a haplotype block modulating the profile of the multimodal FFA phenotype. In our sample, the 4 SNPs were in strong linkage disequilibrium with all D′ > 0.90, and we reproduced the 3 haplotypes: (1) a high enzymatic activity (HEA) haplotype (rs6269/G-rs4633/C-rs4818/G-rs4680/G) with a frequency of 0.328, (2) an intermediate enzymatic activity (MEA) haplotype (rs6269/A-rs4633/T-rs4818/C-rs4680/A) with a frequency of 0.239, and (3) a low enzymatic activity (LEA) haplotype (rs6269/A-rs4633/C-rs4818/C-rs4680/G) with the highest frequency of 0.389 (Table 1). Diplotype estimation revealed 34 homozygous HEA (low dopamine availability) haplotype carriers, 143 heterozygous HEA (intermediate dopamine availability) haplotype carriers, and 139 HEA haplotype non-carriers (high dopamine availability)^33^ in our sample (Table 1). A two-way ANOVA was carried out on PLS1-FFA scores by haplotype and hemisphere (Fig. 2). The interaction between the effects of haplotype and hemisphere on PLS1-FFA scores was not significant [F(2,626) = 0.252, p = 0.777]. The main effect of haplotype was significant [F(2,626) = 9.436, p < 0.001, partial eta^2^ = 0.031, power = 0.809], but the main effect of hemisphere was not significant [F(1,626) = 0.003, p = 0.958]. Tukey’s HSD post hoc tests showed that COMT heterozygous-HEA carriers had significant larger PLS1 rFFA-MRI scores than COMT homozygous HEA carriers (mean difference = 0.011, 95%CI [0.002, 0.021], *p* = 0.005, *p*_FDR_ = 0.017) and non-HEA carriers (mean difference = 0.010, 95%CI [0.004, 0.017], *p* < 0.001, *p*_FDR_ < 0.001). Thus, participants with the COMT heterozygous-HEA-haplotype (intermediate DA degrading enzymatic activity therefore an intermediate level of DA) showed increased FS and decreased rFFA-fALFF (positive FFA scores of PLS1), whereas participants with the COMT homozygous HEA haplotype (high DA degrading enzymatic activity therefore a low level of DA) and HEA non-carriers (low DA degrading enzymatic activity therefore a high level of DA) showed decreased FS and increased rFFA-fALFF (negative FFA scores of PLS1).

**Table 1:** Demographic Information, Frequency of *COMT* Polymorphism, and Face Recognition Performance in *COMT* Genotype and Haplotype Groups.

SNPs	Genotype	GF	N (Male)	Age years	Raven score	Face accuracy	Face sensitivity	Flower accuracy	Flower sensitivity
rs6269_G	A/A	0.427	146 (55)	20.3 (1.0)	24.9 (6.8)	76.3 (8.8)	1.6 (1.0)	77.2 (9.7)	2.1 (1.4)
	A/G	0.462	155 (65)	20.5 (0.8)	25.6 (6.0)	75.8 (9.1)	1.7 (1.1)	77.8 (8.3)	2.1 (1.3)
	G/G	0.111	37 (14)	20.6 (1.2)	25.7 (3.7)	75.4 (10.7)	1.7 (1.2)	77.5 (8.6)	2.0 (1.2)
rs4633_T	C/C	0.557	187 (78)	20.5 (1.0)	26.0 (5.1)^*^	75.7 (10)	1.7 (1.2)	77.7 (8.9)	2.1 (1.3)
	C/T	0.377	125 (45)	20.3 (0.9)	24.3 (6.3)^*^	76.7 (8.0)	1.6 (0.9)	77.3 (9.0)	2.0 (1.2)
	T/T	0.066	25 (11)	20.2 (1.0)	24.8 (6.5)	74.9 (10)	1.6 (1.1)	77.3 (10)	2.1 (1.4)
rs4818_G	C/C	0.434	143 (53)	20.3 (1.0)	24.8 (6.9)	76.2 (9.0)	1.6 (1.0)	77.7 (9.8)	2.1 (1.4)
	C/G	0.456	148 (63)	20.5 (0.8)	25.6 (5.0)	75.9 (9.1)	1.7 (1.1)	77.6 (8.4)	2.1 (1.3)
	G/G	0.111	35 (14)	20.7 (1.3)	25.9 (3.5)	75.7 (9.1)	1.7 (1.2)	77.8 (8.6)	2.1 (1.2)
rs4680_A	G/G	0.582	193 (78)	20.5 (1.0)	26.0 (5.2)^*^	75.5 (9.7)	1.7 (1.1)	77.9 (8.8)	2.1 (1.3)
	A/G	0.358	121 (46)	20.3 (0.9)	24.4 (6.4)^*^	82.7 (8.2)	1.6 (0.9)	76.9 (9.2)	2.1 (1.3)
	A/A	0.060	21 (9)	20.5 (0.9)	25.2 (5.7)	76.8 (8.9)	1.7 (1.1)	79.0 (8.7)	2.2 (1.3)
Haplotype combination rs6269-rs4633-rs4818-rs4680	Homozygous-HEA (GCGG/GCGG)	0.108	34 (13)	20.6 (1.1)	25.7 (3.4)	75.7 (10.5)	1.7 (1.2)	77.7 (8.8)	2.1 (1.2)
	Heterozygous-HEA	0.453	143 (57)	20.5 (0.9)	25.6 (5.1)	76.0 (9.1)	1.7 (1.1)	77.9 (8.3)	2.1 (1.3)
	Non-carrier	0.439	139 (54)	20.3 (1.0)	24.8 (6.5)	76.1 (8.9)	1.6 (1.0)	77.7 (9.6)	2.1 (1.4)

GF, frequency of genotype; N, number of subjects. Allele frequencies in this sample were very similar to those in the Han Chinese sample from the HapMap dataset (HapMap Data Release 27 Phase II + III). Standard deviation (SD) were put in the parentheses.

^*^Participants with the rs4633 CC genotype showed higher Raven scores than those with the rs4633 CT genotype (*p* = 0.013, uncorrected); participants with the rs4680 GG genotype showed higher Raven scores than those with the rs4633 AG genotype (*p* = 0.016, uncorrected).

### Face-specific recognition ability is associated with a profile of FFA functional activity

**Figure 3:**
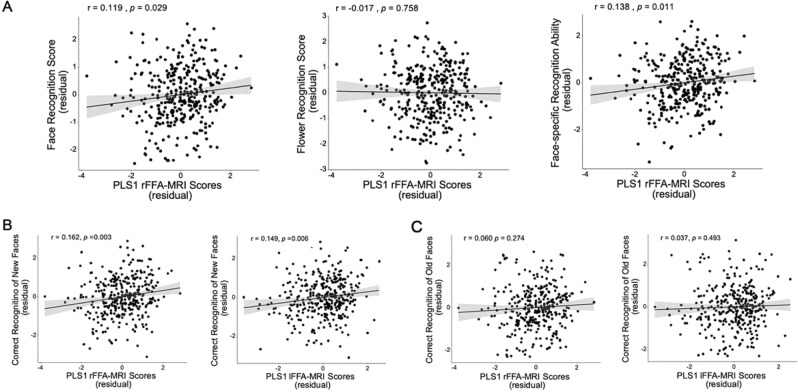
(**A**) The face-specific recognition ability and face recognition accuracy, but not the flower recognition score, were positively correlated with the PLS1 3-modal MRI scores of the right FFA. (**B**) The associations between the new-face recognition ability and the bilateral PLS1 FFA-MRI scores were significant. (**C**) The associations between the old-face recognition ability and the bilateral PLS1 FFA-MRI scores were not significant. Age, sex, and Raven scores were treated as nuisance covariates in the analyses.

To investigate whether the functional rFFA-SNP association contributes to the FRA, we correlated the face recognition accuracy score (hit rate + correct rejection rate) and flower recognition accuracy score with the PLS1 rFFA-MRI scores and the PLS1 COMT scores, respectively. The results showed that face recognition accuracy was positively correlated with the PLS1 rFFA-MRI scores (r = 0.119, uncorrected *p* = 0.029; left Panel of Fig. 3A) when sex, age, and Raven scores were treated as nuisance covariates. No significant association was found between the flower recognition accuracy and the PLS1 rFFA-MRI scores (middle panel of Fig. 3A). Moreover, the association between the PLS1 rFFA-MRI profile and the face recognition accuracy was significantly greater than that between the PLS1 rFFA-MRI profile and the flower recognition accuracy (z = 2.94, two-tailed *p* = 0.003, Cohen’*d* = 0.4, 95%CI = [0.06,0.28]), suggesting that the COMT-modulated rFFA-MRI profile was more relevant to the face recognition ability than to the flower recognition ability. Further, the association between the FRA (the normalized residual of the face recognition score after regressing out the flower recognition score) and the PLS1 rFFA-MRI scores was significant (r = 0.138, uncorrected *p* = 0.011, 95%CI [0.01,0.22], power = 0.721) when sex, age, and Raven scores were treated as nuisance covariates (right panel of Fig. 3A). The results suggested that a profile of increased FS and decreased fALFF in the right FFA might be associated with a better face-specific recognition ability. In the left FFA, although the association between the PLS1 lFFA-MRI scores and the FRA was not significant (r = 0.094, *p* = 0.084) (panel C of Supplementary Fig. S4), the associations of the FRA with the PLS1 lFFA-MRI scores and the PLS1 rFFA-MRI scores did not differ significantly (z = 0.966, *p* = 0.334), suggesting that the COMT-modulated bilateral FFA-MRI profile might have similar association patterns with the FRA. To make our results comparable to those in previous studies using the signal detection theory, we calculated the sensitivity index (d’) for face recognition and flower recognition. No significant correlations were found between face sensitivity (d’) and the PLS1 FFA-MRI score (Supplementary Table 3), suggesting that the COMT-profile modulated PLS1-FFA-MRI profile might be specific, but not sensitive enough, to face recognition.

Finally, because the FFA has been demonstrated to be involved in the process of recognizing familiar and unfamiliar faces^44–47^, we examined whether there is a difference in modulation of the COMT genotypic profile between old (familiar) face recognition and new (relatively unfamiliar) face recognition. To control the effect of response bias on the accuracy^48^, we calculated the response bias [c = −Z(hit rate) + Z(false alarm rate)]^49^ and regressed the bias out of the hit rate and correct rejection rate to obtain the old-face recognition score and new-face recognition score. A significant association was found between the new-face recognition score and the bilateral PLS1 FFA-MRI scores (rFFA: r = 0.162, uncorrected *p* = 0.003; lFFA: r = 0.149, uncorrected *p* = 0.006; Fig. 3B), but not between the old-face recognition score and the bilateral PLS1 FFA-MRI scores (Fig. 3C, Supplementary Table S3 and Fig. S5) when controlling for age, sex, and Raven scores. Moreover, the association between the PLS1 FFA-MRI profile and the new-face recognition score was significantly greater than that between the PLS1 FFA-MRI profile and the old-face recognition score (rFFA: z = 2.70, two-tailed *p* = 0.007, Cohen’d = 0.33, 95%CI = [0.08, 0.39]; lFFA: z = 2.95. two-tailed *p* = 0.003, Cohen’d = 0.36, 95%CI = [0.13,0.43]), suggesting that the COMT-modulated FFA-MRI profile was more sensitive to the new-face recognition ability than to the old-face recognition ability.

## Discussion

In the present study, we explored the neural basis of the heritability of face recognition by investigating associations between 12 COMT genotypes and 3-modal FFA-MRI phenotypes. The results revealed that a profile of face-selective responses and resting-state neural fluctuations of the bilateral FFA were modulated by a profile of *COMT* polymorphisms. Participants with the *COMT* heterozygous-HEA-haplotype showed higher PLS1 FFA-MRI scores, which represented an increased FS and a decreased fALFF and were associated with a better face-specific recognition ability, than those with the *COMT* homozygous HEA haplotype and HEA non-carrier, suggesting that participants with an appropriate (intermediate) level of dopamine in the bilateral FFA may have better face-specific recognition ability. Our study provides empirical evidence of genetic modulation of a functionally-specialized cortical region in humans.

Previous studies have found that participants with higher face selectivity in the FFA have higher face recognition ability^2,3^, and this association is domain-specific^3^. In this study, we revealed that a profile (linear combination) of higher face selectivity BOLD responses, as well as lower resting-state activities in the bilateral FFA, was associated with a higher face recognition (especially the new-face recognition) ability, and the association could not be accounted for by FFA responses to objects such as flowers, suggesting that the face-specific recognition ability might be modulated by a certain covariation pattern of resting-state and task-state activity in the FFA, which was modulated by a certain profile of the COMT polymorphisms.

The *COMT* Val/Met (rs4680) polymorphism has been suggested to play a role in face perception^15–19,22^. Our study extends previous findings by identifying a neural site where *COMT* affects face recognition. Specifically, we investigated a broader modulation pattern of *COMT* genetic effects on the FFA by including multiple SNPs. We found that individuals with the heterozygous HEA haplotype exhibited higher face-selective responses but smaller fALFF. In contrast, individuals with the homozygous HEA haplotype or HEA non-carriers had lower face-selective responses and larger fALFF. Because *COMT* encodes catechol-O-methyltransferase, a protein that degrades catecholamine neurotransmitters and modulates dopamine metabolism^20^, neural activity (including the face-recognition-task-state and resting-state activities) in the FFA is likely modulated by changes in dopamine. Moreover, we found that an intermediate level of dopamine in the FFA was associated with higher face-selective responses (in the task state) and smaller fALFF (in the resting state), and the profile of higher face-selective responses and lower fALFF in the FFA was associated with better face-specific recognition ability. Thus, an intermediate level of dopamine in the FFA is likely beneficial for behavioral performance in face recognition. That is to say, participants with an appropriate level of dopamine (i.e., the heterozygous HEA haplotype carries) in the FFA possess a functional characteristic profile of higher face-selective responses and lower intrinsic FFA activities, therefore, they might have better face-specific recognition ability.

Previous studies have rarely reported the direct impact of COMT polymorphisms on resting-state FFA activity in healthy people. Some studies focusing on the interaction effect between the *COMT* rs4680 and other dopamine-function related generic variants imply that dopamine-related neurons may affect the baseline brain resting state activity by synchronizing the low-frequency oscillation^50^. Moreover, dopamine could stabilize (keep the balance between facilitating and suppressing) neural signaling in brain networks responsible for behavior-stimuli oriented attention^51,52^. Thus, an appropriate level of dopamine may improve the functional segregation-integration of a functional-specialized region by maintaining excitation-inhibition stability of neural signal transduction to ensure that individuals with the HEA heterozygous haplotype have a relatively higher rFFA activation in face recognition tasks (which has been found to be related to a better face-specific recognition ability^2,3^), as well as a relatively lower rFFA activity during resting state (which was inferred from previous research that lower metabolism in the right temporal-parietal cortex is also associated with better cognitive reserve and memory ability in aging people^53^). This is consistent with previous studies demonstrating associations between dopamine hyper- or hypo-function in cortical or subcortical regions and cognitive dysfunctions^54–56^. Therefore, it would be interesting to examine the level of dopamine in the FFA in individuals with developmental prosopagnosia^10,57–59^, who show specific deficits in face recognition with unclear genetic basis^60^ and those with mental disorders who might have disturbances in COMT modulation of face processing^16,18,21,23^.

Our study found that the 4 COMT-polymorphisms-modulated PLS1 rFFA-MRI profile mainly regulated the recognition ability of new faces. Although previous studies have revealed that it takes more time and is more likely to make mistakes to recognize faces of different identities than faces of same identities^47^, there seems to be no difference in the increase of neural response activity of the FFA when recognizing familiar and unfamiliar faces^44,47^. Compared with recognizing unfamiliar faces, recognizing familiar faces involves more activity in the anterior and mid-fusiform gyrus and extended face areas^44,45^. In our study, the stimuli were not familiar to the subjects, and the social and emotional information involved in the face stimuli was relatively sparse; therefore, the recognition difference between the old face and the new face might be mainly reflected in the familiarity and unfamiliarity of the facial features. We found that the COMT-polymorphisms modulated PLS1 rFFA-MRI profile was sensitive to the recognition of new faces, but not to that of the old faces. The findings may be related to the fact that the novel signals can trigger the dopaminergic metabolic system^55^ in the task-related brain areas and enhance perception^61,62^.

The FFA is sensitive to the static or invariant properties of faces^63^. The posterior superior temporal sulcus (pSTS) is responsible for dynamic features^63,64^ and sensitive to emotional information^65–67^ from faces, and the prefrontal-amygdala circuit is involved in social and emotional regulation in face processing^23^. In fact, face perception as a whole is a network involving multiple brain regions^8,9^. Studies have found that the COMT Met allele is related to the modulation of neural substrates of negative face emotional bias^22,23^. Therefore, in future studies, it would be interesting to evaluate the role of multiple COMT polymorphisms in the core face network and its relation with face-specific recognition ability and face-emotion recognition.

This study has some limitations. First, although we have adopted multiple strategies to improve the statistical power, the findings still need to be validated using data from other cohorts of participants. Second, measurements for the face recognition ability and the general mental ability are limited to the face memory test and the Raven test, respectively. More multivariate measurements could be introduced in the future to improve the reliability of the individual face recognition ability and general mental ability, and further elaborate the specificity of the effect of COMT on the face recognition ability. Third, all participants had the same ethnicity (i.e., Han Chinese); therefore, how the ethnicity of the individual participants affected the results needs to be examined using data from multiple ethnicities in the future. Fourth, the Caucasian children’ faces were used as stimuli to define the FFA in Han Chinese participants. As demonstrated in previous studies, there is an other-race effect in face recognition; therefore, how our findings are affected by stimulus attributes still needs to be investigated in further studies.

In conclusion, our study demonstrated that the *COMT* gene affected both resting- and task-state neural activity in the FFA through rs6269-rs4633-rs4818-rs4680 haplotypes, and the COMT-polymorphisms-modulated FFA was associated with face-specific recognition ability. The present findings provide a basis for the behavioral observation of face-specific recognition heritability and the approach used in this study might be s a feasible method for exploring and validating the neural basis of heritable behavioral performance. In addition, this study may inform the genetic etiology of developmental prosopagnosia and other mental disorders showing abnormities in face processing.

## Methods

### Participants

Three hundred and sixty-two Han Chinese college students (mean age, 20.47 ± 0.96 years; 144 males) were recruited from Beijing Normal University (BNU) in Beijing, China through campus advertisement. Ethnicity was determined by the ethnic information displayed on the ID card of the participants. All participants had normal or corrected-to-normal vision, and none of them reported a history of neurological or psychiatric disorders. All the experimental protocol and procedures were approved by the Committee for Protecting Human and Animal Subjects of the Faculty of Psychology at Beijing Normal University. All experiments of the study were conducted in accordance with the relevant guidelines and regulations of Beijing Normal University’s Institutional Review Board (Human Subjects Division). All participants gave written informed consent prior to their participation in this study.

### MRI Data acquisition

Functional and structural MRI was performed at the BNU Imaging Center for Brain Research on a Siemens 3 T scanner (MAGENTOM Trio, a Tim system) with a 12-channel phased-array head coil. Resting-state fMRI was acquired using a T2*-weighted gradient-echo echo-planar-imaging (GRE-EPI) sequence (repetition time [TR] = 2000 ms, echo time [TE] = 30 ms, flip angle = 90°, number of slices = 33, voxel size = 3.125 × 3.125 × 3.6 mm^3^). Resting-state scanning lasted for 8 min and consisted of 240 contiguous volumes. Participants were instructed to relax and remain still with their eyes closed during resting state scanning^9^. A GRE-EPI sequence was also used to acquire task-state fMRI (TR = 2000 ms, TE = 30 ms, number of slices = 30, voxel size = 3.125 × 3.125 × 4.8 mm^3^). A high-resolution structural T1-weighted image was acquired with a 3D magnetization-prepared rapid acquisition gradient echo (MP-RAGE) sequence (TR = 2530 ms, TE = 3.39 ms, inversion time = 1100 ms, flip angle = 7°, matrix = 256 × 256, number of slices = 128, voxel size = 1 × 1 × 1.33 mm^3^). Earplugs were used to attenuate scanner noise, and a foam pillow and extendable padded head clamps were used to restrain head motion^3^. None of the participants were excluded due to excessive head motion (>2 mm in translation or 2° in rotation) during MRI scanning.

### MRI Data processing

#### Structural MRI

Voxel-based morphometry (VBM) was performed using the SPM8 (Statistical Parametric Mapping, Wellcome Department of Imaging Neuroscience, London, UK). First, unified segmentation in SPM8 was used to segment T1-weighted anatomical images into gray matter (GM), white matter (WM), and cerebral spinal fluid (CSF). Second, the Diffeomorphic Anatomical Registration through Exponential Lie algebra (DARTEL) registration method^68^ was used to build a study-specific GM template and normalize individual GM images into the template. Third, GM voxel values were modulated by multiplying the Jacobian determinants derived from the normalization procedure in order to preserve the tissue volume of each structure. Fourth, modulated GM images were smoothed using an 8-mm full width at half maximum (FWHM) isotropic Gaussian kernel. Finally, the modulated images were masked using absolute masking with a threshold of 0.2 to exclude noisy voxels. Masked-modulated GM images were used for further statistical analyses^3,69^.

#### Task-state fMRI

A dynamic face localizer was used to define the face-selective area and derive FS^3,63^. Movie clips of close-up faces of 7 Caucasian children (filmed when children were dancing or playing), objects (moving toys), scenes (moving view of a suburb, canyons, or tunnels), and scrambled objects (constructed by scrambling each frame of the object movie clips) were included to examine category selectivity in the ventral visual cortex^63^. Participants were instructed to passively view movie clips during scanning. Because Adults’ FFA have been effectively identified by children’ faces in previous studies^3,5,63,65,67^ and the FFA is sensitive to the invariant properties of faces, but not to the dynamic or emotional information of faces^65–67,70^, we expected that the dynamic children’ faces would accurately and unbiasedly localize the FFA in adults.

**Figure 4:**
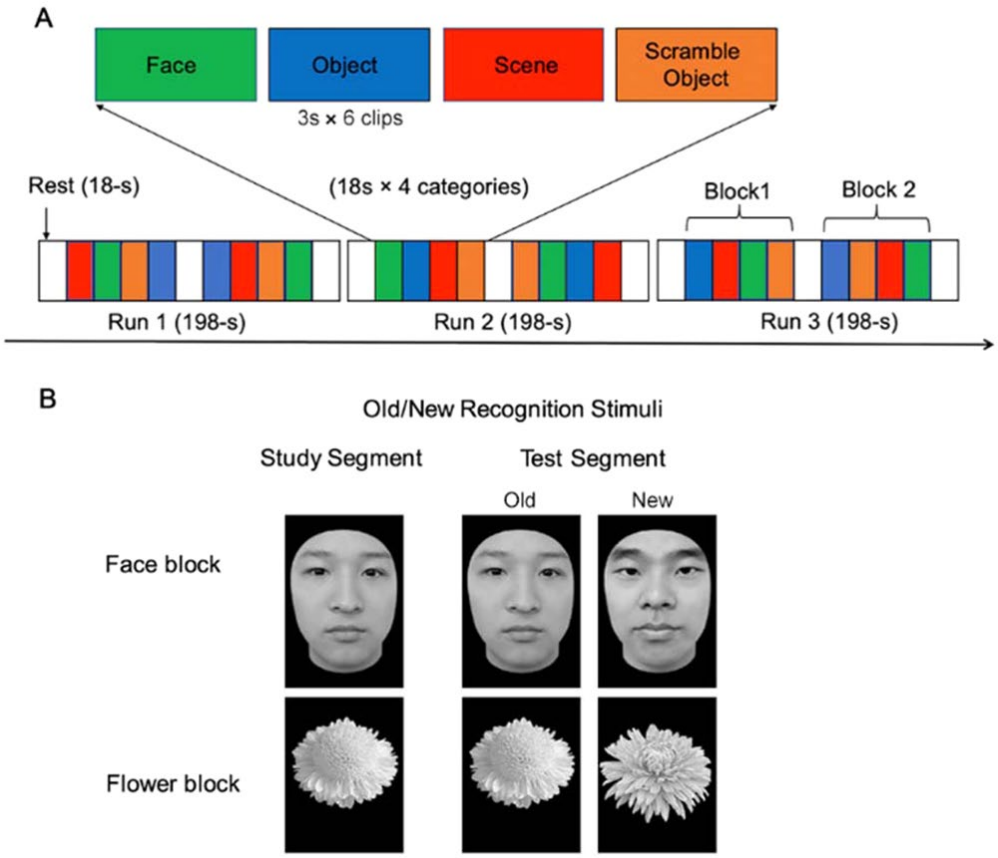
(**A**) Illustration of scanning sequence for the functional MRI and localization of dynamic face, object, scene, and scramble. (**B**) Example stimuli and trial types in the old/new recognition task. Participants studied a single image (either a face or a flower), and they were then shown a series of individual images of the corresponding type and asked to indicate which of the images had been shown in the study segment.

In total, participants completed three 198-s runs. Each run consisted of 2 block sets intermixed with 3 18-s rest blocks at the beginning, middle, and end of the run. Each block set contained four 18-s (including six 3-s movie clips) blocks corresponding to 4 stimulus categories. The order of category blocks in each run was palindromic and randomized across runs (Fig. 4A).

Functional images were analyzed with the FMRI Expert Analysis Tool version 5.98, part of FMRIB’s Software Library (www.fmrib.ox.ac.uk/fsl). Preprocessing included head-motion correction, brain extraction, spatial smoothing (Gaussian kernel; FWHM = 6 mm), grand-mean intensity normalization, and high-pass temporal filtering (120-s cutoff). A first-level analysis was performed separately on each run for each participant using FMRIB’s Improved Linear Model with a local autocorrelation correction. A boxcar kernel was convolved with a gamma hemodynamic response function and its temporal derivative was used to model BOLD signal. Six parameters from motion-correction were also included in the model as regressors of no interest to account for the effect of head movement. A second-level fixed-effect analysis was conducted to combine all runs. Specifically, the parameter (i.e., beta) image from the first-level analysis was first aligned to the individual’s structural images using FMRIB’s linear image registration tool with 6 degrees of freedom and then warped to the MNI152 template using FMRIB’s nonlinear image registration tool with default parameters. The spatially normalized parameter images (resampled to 2-mm isotropic voxels) were then summarized across all runs using a fixed-effect model^3,9,69^. In this study, statistical images from the contrast between faces and objects were used to define the right fusiform face area and derive its face selectivity (FS), which was calculated as the average z-score (faces vs. objects) across all voxels in the defined face-selective region.

#### Resting-state fMRI

Resting-state functional images were preprocessed with a procedure similar to task-state fMRI. Motion correction parameters, mean signals of the CSF and the WM, and the first derivatives of these signals were regressed out to remove the signal fluctuations caused by head motion, cardiac cycle and respiration. Then, the fractional amplitude of low-frequency fluctuations (fALFF), which is defined as the fractional sum of the amplitudes within the low frequency range divided by the sum of amplitude across the entire frequency range (0–0.25 Hz)^71^, was calculated for each GM voxel. Participant-level voxel-wise fALFF maps were further standardized by subtracting the mean whole-brain voxel-wise fALFF from the participant-level voxel-wise fALFF and dividing it by the standard deviation. Finally, the standardized fALFF was normalized to the MNI152 space with the same normalization procedure as that used in the task-state fMRI analysis.

#### Identification of fusiform face area

A threshold of Z > 2.58 (one-tailed p < 0.005, uncorrected) was used to define the bilateral FFA based on the random-effect group-level Z-statistic image for faces vs. objects^3^. Specifically, all contiguous voxels that surpassed the threshold within the posterior fusiform area (defined by the anatomical automatic labeling [AAL] template; http://www.gin.cnrs.fr/AAL) were defined as the FFA (right panel of Figs. 1A and S4).

### Genotyping

Genomic DNA was extracted from peripheral blood samples of each subject using QuickGene-Mini80 equipment and the QuickGene DNA whole blood kit S (Fujifilm). Sixty-four cognition-related candidate SNPs (including the four COMT polymorphisms of rs6269, rs4633, rs4818, and rs4680 examined in this study; please refer to Supplementary Table S1 for details of other SNPs) were automatically genotyped using a customized 64 TaqManOpenArray GT Kit (Applied Biosystems; Foster City, CA, USA). Quality control was performed using PLINK software version 1.07^72^. All the 4 COMT SNPs met the following criteria: genotyping call rate >0.95, minor allele frequency (MAF) > 0.05, and Hardy–Weinberg equilibrium (HWE) P > 0.05. Participants were excluded from analyses if they had a missing genotype rate of >25% (i.e., the participants who had one of the four COMT polymorphisms missing). Twenty-four participants were excluded, and the remaining 338 participants (mean age, 20.45 ± 0.96 years; 135 males) were included in the data analyses. Allele frequencies in this sample were very similar to those in the Han Chinese sample from the HapMap dataset (HapMap Data Release 27 Phase II + III).

### Face recognition task

We used an old/new cognitive memory task to measure the face-specific recognition ability (FRA). There was a face block (containing grayscale face pictures of adult Chinese faces with the external contours removed) and a flower block (containing grayscale pictures of common flowers) in this task (face and flower stimuli were shown in Fig. 4B) that were counterbalanced across participants. Each block consisted of one study segment and one test segment. In the study segment, each stimulus (picture) was presented twice (1 second each time), with an inter-stimulus interval of 0.5 seconds. Then, in the test segment, half of the studied images were randomly intermixed with new images from the same category. For each stimulus picture, participants were instructed to indicate whether the image had been shown in the study segment. For a participant, a face recognition accuracy score was calculated as the average proportion of hits and correct rejections for faces, and an object (flower) recognition accuracy score was calculated as the average proportion of hits and correct rejections for flowers. The FRA was calculated as the normalized residual of the face recognition score after regressing out the flower recognition score^3^. D’ (sensitivity index)^48^ was also calculated for face recognition and flower recognition.

### Statistical analyses

The PLS correspondence analysis (PLSCA) was conducted using the R Package ExPosition (https://cran.r-project.org/web/packages/ExPosition/). PLS is particularly suitable for identifying associations between 2 sets of variables, especially when variables of each set are highly interdependent or multi-collinear^38–40,73,74^. The PLS approach maximizes the covariance between a set of variables (a linear combination of X measures) and another set of variables (a linear combination of Y measures). More specifically, the cross-correlation matrix between measures of SNPs and measures of brain-MRI was first computed. Then, a set of paired latent variables (LVs), which were uncorrelated (orthogonal) with each other and ordered by the explained covariance of brain and SNP measures, were generated via singular value decomposition. LVs are linearly weighted by the raw variables, and these weights or saliences measure the contribution of each raw variable to the LV^73^. Finally, the significance of the variance of singular values was tested by comparison with the distribution of variance arising from random permutation tests (1000 repetitions), and a bootstrap procedure (500 times) was used to test the reliability (i.e, bootstrap ratio, BSR) of each variable in the significant PLS component^40,73^.

#### SNPs-FFA association analysis

The SNPs-FFA multivariate association analysis was used to examine whether specific patterns of COMT polymorphisms contributed to the multimodal FFA-MRI phenotypes. First, GM, fALFF, and FS within the right FFA and the left FFA ROI were extracted for each participant. Then, each SNP was coded in accordance with genotypic (codominant) models^37,75^: each SNP was coded into 3 genotypes (e.g., AA, AG, or GG) as categorical variables, and each variable was weighted according to the information it provided. The weight of a variable was defined as the inverse of its relative frequency because a rare variable provides more information than does a frequent variable^37,75^. This fully categorical coding scheme allows us to detects linear and nonlinear relationships within (e.g., genotypic) and between datasets^37^. Thus, treating each genotype as a level of a categorical variable is suitable for genetic association studies, especially when the inheritance pattern, direction, and/or size of the effect are unknown^37,76^. Accordingly, a matrix of measures of 12 *COMT* genotypes estimated in the 338 participants and a matrix of measures of 3-modal FFA-MRI phenotype estimated in the 338 participants were entered into the PLS analysis.

#### Haplotype analysis

A haplotype block analysis^77^ was conducted to investigate how SNPs in strong linkage disequilibrium work as blocks to modulate FFA phenotypes. Haplotype blocks of SNPs affecting the FFA phenotype were assessed and illustrated using Haploview software version 4.2 (https://sourceforge.net/projects/haploview/) with the solid spine of the linkage disequilibrium method and parameter 0.80. Associations of haplotypes with the FFA were examined using R software version 3.5.2 with generalized linear models.

## Supplementary information

Supplementary Information

## References

[1] Crouch DJM (2018). Genetics of the human face: Identification of large-effect single gene variants. Proc. Natl Acad. Sci. USA.

[2] Furl N, Garrido L, Dolan RJ, Driver J, Duchaine B (2011). Fusiform gyrus face selectivity relates to individual differences in facial recognition ability. J. Cogn. Neurosci..

[3] Huang L (2014). Individual differences in cortical face selectivity predict behavioral performance in face recognition. Front. Hum. Neurosci..

[4] Shakeshaft NG, Plomin R (2015). Genetic specificity of face recognition. Proc. Natl Acad. Sci. USA.

[5] Zhen Z (2015). Quantifying interindividual variability and asymmetry of face-selective regions: a probabilistic functional atlas. Neuroimage.

[6] Zhu Q (2010). Heritability of the Specific Cognitive Ability of Face Perception. Curr. Biol. Cb.

[7] Kanwisher N, McDermott J, Chun MM (1997). The fusiform face area: a module in human extrastriate cortex specialized for face perception. J. Neurosci..

[8] Ishai A (2008). Let’s face it: it’s a cortical network. Neuroimage.

[9] Wang X (2016). The Hierarchical Structure of the Face Network Revealed by Its Functional Connectivity Pattern. J. Neurosci..

[10] Zhao Y, Zhen Z, Liu X, Song Y, Liu J (2017). The neural network for face recognition: Insights from an fMRI study on developmental prosopagnosia. Neuroimage.

[11] Pinel P (2015). Genetic and Environmental Influences on the Visual Word Form and Fusiform Face Areas. Cereb. Cortex.

[12] Polk TA, Park J, Smith MR, Park DC (2007). Nature versus nurture in ventral visual cortex: a functional magnetic resonance imaging study of twins. J. Neurosci..

[13] Dickie EW (2014). Global genetic variations predict brain response to faces. PLoS Genet..

[14] Brown AA (2012). Genetic variants affecting the neural processing of human facial expressions: evidence using a genome-wide functional imaging approach. Transl. Psychiatry.

[15] Doi H, Nishitani S, Shinohara K (2015). Association between catechol-O-methyltransferase Val(1)(5)(8)Met polymorphism and configural mode of face processing. Neurosci. Lett..

[16] Soeiro-de-Souza MG (2012). COMT Met (158) modulates facial emotion recognition in bipolar I disorder mood episodes. J. Affect. Disord..

[17] Rostami HN (2017). COMT genotype is differentially associated with single trial variability of ERPs as a function of memory type. Biol. Psychol..

[18] Hidding E, Swaab H, de Sonneville LM, van Engeland H, Vorstman JA (2016). The role of COMT and plasma proline in the variable penetrance of autistic spectrum symptoms in 22q11.2 deletion syndrome. Clin. Genet..

[19] Lamb YN, McKay NS, Singh SS, Waldie KE, Kirk IJ (2016). Catechol-O-methyltransferase val(158)met Polymorphism Interacts with Sex to Affect Face Recognition Ability. Front. Psychol..

[20] Gohier B (2014). Genetic modulation of the response bias towards facial displays of anger and happiness. Eur. Psychiatry.

[21] Williams LM (2010). COMT Val(108/158)Met polymorphism effects on emotional brain function and negativity bias. Neuroimage.

[22] Surguladze SA (2012). Interaction of catechol O-methyltransferase and serotonin transporter genes modulates effective connectivity in a facial emotion-processing circuitry. Transl. Psychiatry.

[23] Lischke A (2018). COMTVal158Met Genotype Affects Complex Emotion Recognition in Healthy Men and Women. Front. Neurosci..

[24] Domschke K (2012). Catechol-O-methyltransferase gene variation: impact on amygdala response to aversive stimuli. Neuroimage.

[25] Schacht JP (2016). COMT val158met moderation of dopaminergic drug effects on cognitive function: a critical review. Pharmacogenom. J..

[26] Geller S, Wilhelm O, Wacker J, Hamm A, Hildebrandt A (2017). Associations of the COMT Val 158 Met polymorphism with working memory and intelligence – A review and meta-analysis. Intelligence.

[27] Tang C (2019). Meta-Analysis of the Effects of the Catechol-O-Methyltransferase Val158/108Met Polymorphism on Parkinson’s Disease Susceptibility and Cognitive Dysfunction. Front. Genet..

[28] Barnett JH, Scoriels L, Munafo MR (2008). Meta-analysis of the cognitive effects of the catechol-O-methyltransferase gene Val158/108Met polymorphism. Biol. Psychiatry.

[29] Zille P, Calhoun VD, Wang YP (2018). Enforcing Co-Expression Within a Brain-Imaging Genomics Regression Framework. IEEE Trans. Med. Imaging.

[30] Vertes PE (2016). Gene transcription profiles associated with inter-modular hubs and connection distance in human functional magnetic resonance imaging networks. Philos. Trans. R. Soc. Lond. B Biol. Sci..

[31] Whitaker KJ (2016). Adolescence is associated with genomically patterned consolidation of the hubs of the human brain connectome. Proc. Natl. Acad. Sci. USA.

[32] Diatchenko L (2005). Genetic basis for individual variations in pain perception and the development of a chronic pain condition. Hum. Mol. Genet..

[33] Schmack K (2015). Linking unfounded beliefs to genetic dopamine availability. Front. Hum. Neurosci..

[34] Sui J (2010). A CCA+ICA based model for multi-task brain imaging data fusion and its application to schizophrenia. Neuroimage.

[35] Pearlson GD, Liu J, Calhoun VD (2015). An introductory review of parallel independent component analysis (p-ICA) and a guide to applying p-ICA to genetic data and imaging phenotypes to identify disease-associated biological pathways and systems in common complex disorders. Front. Genet..

[36] Calhoun VD, Sui J (2016). Multimodal fusion of brain imaging data: A key to finding the missing link(s) in complex mental illness. Biol. Psychiatry Cogn. Neurosci. Neuroimaging.

[37] Beaton D, Dunlop J, Abdi H (2016). Partial least squares correspondence analysis: A framework to simultaneously analyze behavioral and genetic data. Psychol. Methods.

[38] McIntosh AR, Bookstein FL, Haxby JV, Grady CL (1996). Spatial pattern analysis of functional brain images using partial least squares. Neuroimage.

[39] McIntosh AR, Chau WK, Protzner AB (2004). Spatiotemporal analysis of event-related fMRI data using partial least squares. Neuroimage.

[40] McIntosh AR, Lobaugh NJ (2004). Partial least squares analysis of neuroimaging data: applications and advances. Neuroimage.

[41] Frassle S, Krach S, Paulus FM, Jansen A (2016). Handedness is related to neural mechanisms underlying hemispheric lateralization of face processing. Sci. Rep..

[42] Ma Y, Han S (2012). Functional dissociation of the left and right fusiform gyrus in self-face recognition. Hum. Brain Mapp..

[43] Aguirre-Urreta M, Rönkkö M (2015). Sample Size Determination and Statistical Power Analysis in PLS Using R: An Annotated Tutorial. Commun. Assoc. Inf. Syst..

[44] Visconti di Oleggio Castello M, Halchenko YO, Guntupalli JS, Gors JD, Gobbini MI (2017). The neural representation of personally familiar and unfamiliar faces in the distributed system for face perception. Sci. Rep..

[45] Ramon M, Vizioli L, Liu-Shuang J, Rossion B (2015). Neural microgenesis of personally familiar face recognition. Proc. Natl Acad. Sci. USA.

[46] Liu J (2014). Neural correlates of covert face processing: fMRI evidence from a prosopagnosic patient. Cereb. Cortex.

[47] Davies-Thompson J, Newling K, Andrews TJ (2013). Image-invariant responses in face-selective regions do not explain the perceptual advantage for familiar face recognition. Cereb. cortex.

[48] Lynn SK, Barrett LF (2014). “Utilizing” signal detection theory. Psychological Sci..

[49] Wenger MJ, Rasche C (2006). Perceptual learning in contrast detection: presence and cost of shifts in response criteria. Psychon. Bull. Rev..

[50] Conio, B. *et al*. Opposite effects of dopamine and serotonin on resting-state networks: review and implications for psychiatric disorders. *Mol. Psychiatry*, 10.1038/s41380-019-0406-4 (2019). 10.1038/s41380-019-0406-4 30953003

[51] Carbonell F (2014). Dopamine precursor depletion impairs structure and efficiency of resting state brain functional networks. Neuropharmacol..

[52] Shafiei G (2019). Dopamine Signaling Modulates the Stability and Integration of Intrinsic Brain Networks. Cereb. cortex.

[53] Bastin C (2012). Cognitive reserve impacts on inter-individual variability in resting-state cerebral metabolism in normal aging. Neuroimage.

[54] Fernandez, M., Mollinedo-Gajate, I. & Penagarikano, O. Neural circuits for social cognition: Implications for autism. *Neuroscience*, 10.1016/j.neuroscience.2017.07.013 (2017). 10.1016/j.neuroscience.2017.07.013 28729065

[55] Howie B, Fuchsberger C, Stephens M, Marchini J, Abecasis GR (2012). Fast and accurate genotype imputation in genome-wide association studies through pre-phasing. Nat. Genet..

[56] Paval, D. A Dopamine Hypothesis of Autism Spectrum Disorder. *Dev Neurosci*, 10.1159/000478725 (2017). 10.1159/000478725 28750400

[57] Zhao Y (2016). Altered spontaneous neural activity in the occipital face area reflects behavioral deficits in developmental prosopagnosia. Neuropsychologia.

[58] Behrmann M, Avidan G (2005). Congenital prosopagnosia: face-blind from birth. Trends Cogn. Sci..

[59] Susilo T, Duchaine B (2013). Advances in developmental prosopagnosia research. Curr. Opin. Neurobiol..

[60] Cattaneo Z (2016). Congenital prosopagnosia is associated with a genetic variation in the oxytocin receptor (OXTR) gene: An exploratory study. Neuroscience.

[61] Schomaker J, Meeter M (2015). Short- and long-lasting consequences of novelty, deviance and surprise on brain and cognition. Neurosci. Biobehav. Rev..

[62] Molas S (2017). A circuit-based mechanism underlying familiarity signaling and the preference for novelty. Nat. Neurosci..

[63] Pitcher D, Dilks DD, Saxe RR, Triantafyllou C, Kanwisher N (2011). Differential selectivity for dynamic versus static information in face-selective cortical regions. Neuroimage.

[64] Pitcher D, Charles L, Devlin JT, Walsh V, Duchaine B (2009). Triple dissociation of faces, bodies, and objects in extrastriate cortex. Curr. Biol..

[65] Pitcher D, Duchaine B, Walsh V (2014). Combined TMS and FMRI reveal dissociable cortical pathways for dynamic and static face perception. Curr. Biol..

[66] Zhang L, Song Y, Liu L, Liu J (2016). Dissociable roles of internal feelings and face recognition ability in facial expression decoding. Neuroimage.

[67] Sliwinska MW, Pitcher D (2018). TMS demonstrates that both right and left superior temporal sulci are important for facial expression recognition. Neuroimage.

[68] Ashburner J (2007). A fast diffeomorphic image registration algorithm. Neuroimage.

[69] Kong F, Hu S, Wang X, Song Y, Liu J (2015). Neural correlates of the happy life: the amplitude of spontaneous low frequency fluctuations predicts subjective well-being. Neuroimage.

[70] Pitcher, D. *et al*. The Human Posterior Superior Temporal Sulcus Samples Visual Space Differently From Other Face-Selective Regions. *Cereb. Cortex*, 10.1093/cercor/bhz125 (2019). 10.1093/cercor/bhz125 PMC7306171 31264693

[71] Zou QH (2008). An improved approach to detection of amplitude of low-frequency fluctuation (ALFF) for resting-state fMRI: fractional ALFF. J. Neurosci. Methods.

[72] Purcell S (2007). PLINK: a tool set for whole-genome association and population-based linkage analyses. Am. J. Hum. Genet..

[73] Krishnan A, Williams LJ, McIntosh AR, Abdi H (2011). Partial Least Squares (PLS) methods for neuroimaging: a tutorial and review. Neuroimage.

[74] Nestor PG (2002). A new statistical method for testing hypotheses of neuropsychological/MRI relationships in schizophrenia: partial least squares analysis. Schizophr. Res..

[75] Beaton D, Filbey F, Abdi H (2013). Integrating Partial Least Squares Correlation and Correspondence Analysis for Nominal. Data..

[76] Lettre G, Lange C, Hirschhorn JN (2007). Genetic model testing and statistical power in population-based association studies of quantitative traits. Genet. Epidemiol..

[77] Barrett JC, Fry B, Maller J, Daly MJ (2005). Haploview: analysis and visualization of LD and haplotype maps. Bioinformatics.

