# Supplementary material for: COMT-Polymorphisms Modulated Functional Profile of the Fusiform Face Area Contributes to Face-Specific Recognition Ability
Source: Sci Rep. 2020 Feb 7;10:2134. doi: 10.1038/s41598-020-58747-4 (PMC7005682; doi:10.1038/s41598-020-58747-4)
Supplement: Supplementary file 1 — Supplementary Information [file 41598_2020_58747_MOESM1_ESM.docx]

***COMT*-Polymorphisms Modulated Functional Profile of the Fusiform Face Area Contributes to Face-Specific Recognition Ability**

Chao Wu^1^, Zonglei Zhen^2*^, Lijie Huang^3^, Taicheng Huang^3^, Jia Liu^2*^

^1^School of Nursing, Peking University Health Science Centre, Beijing, 100191, China

^2^Beijing Key Laboratory of Applied Experimental Psychology, National Demonstration Center for Experimental Psychology Education, Faculty of Psychology, Beijing Normal University, Beijing, 100875, China

^3^State Key Laboratory of Cognitive Neuroscience and Learning and IDG/McGovern Institute for Brain Research, Beijing Normal University, Beijing, 100875, China

*****Address correspondence to Zonglei Zhen and Jia Liu; Room 1415, Main Building, 19 Xinjiekouwai St, Haidian District, Beijing 100875, China. Tel.: +86-10-58806154; Fax: +86-10-58806154. E-mail: [zhenzonglei@bnu.edu.cn](mailto:zhenzonglei@bnu.edu.cn) (Z.Z.); [liujia@bnu.edu.cn](mailto:liujia@bnu.edu.cn) (J.L.).

**Genotyping and Quality Control**

Genomic DNA was extracted from peripheral blood samples of each subject using QuickGene-Mini80 equipment and the QuickGene DNA whole blood kit S (Fujifilm). Sixty-four cognition-related candidate SNPs (including the four COMT polymorphisms of rs6269, rs4633, rs4818, and rs4680 examined in this study; please refer to supplementary Table S1 for details of other SNPs) were automatically genotyped using a customized 64 TaqManOpenArray GT Kit (Applied Biosystems; Foster City, CA, USA). Quality control was performed using PLINK software version 1.07 ^43^. SNPs were removed if they met the following criteria: genotyping call rate < 95% (i.e., for a SNP, if it failed to be genotyped in more than 5% of all participants; in this sample 3 of the 64 SNPs were excluded), Hardy-Weinberg equilibrium (HWE) P < 0.05, minor allele frequency < 5% (5 of the SNPs were excluded). In the current study, all the 4 COMT SNPs met the following criteria: genotyping call rate > 0.95, minor allele frequency (MAF) > 0.05, and Hardy–Weinberg equilibrium (HWE) P > 0.05. Participants were excluded from analyses if they had missing genotype rate > 25% for the 4 COMT polymorphisms (i.e., the participants who had one of the four COMT polymorphisms missing).

**Figure S1**


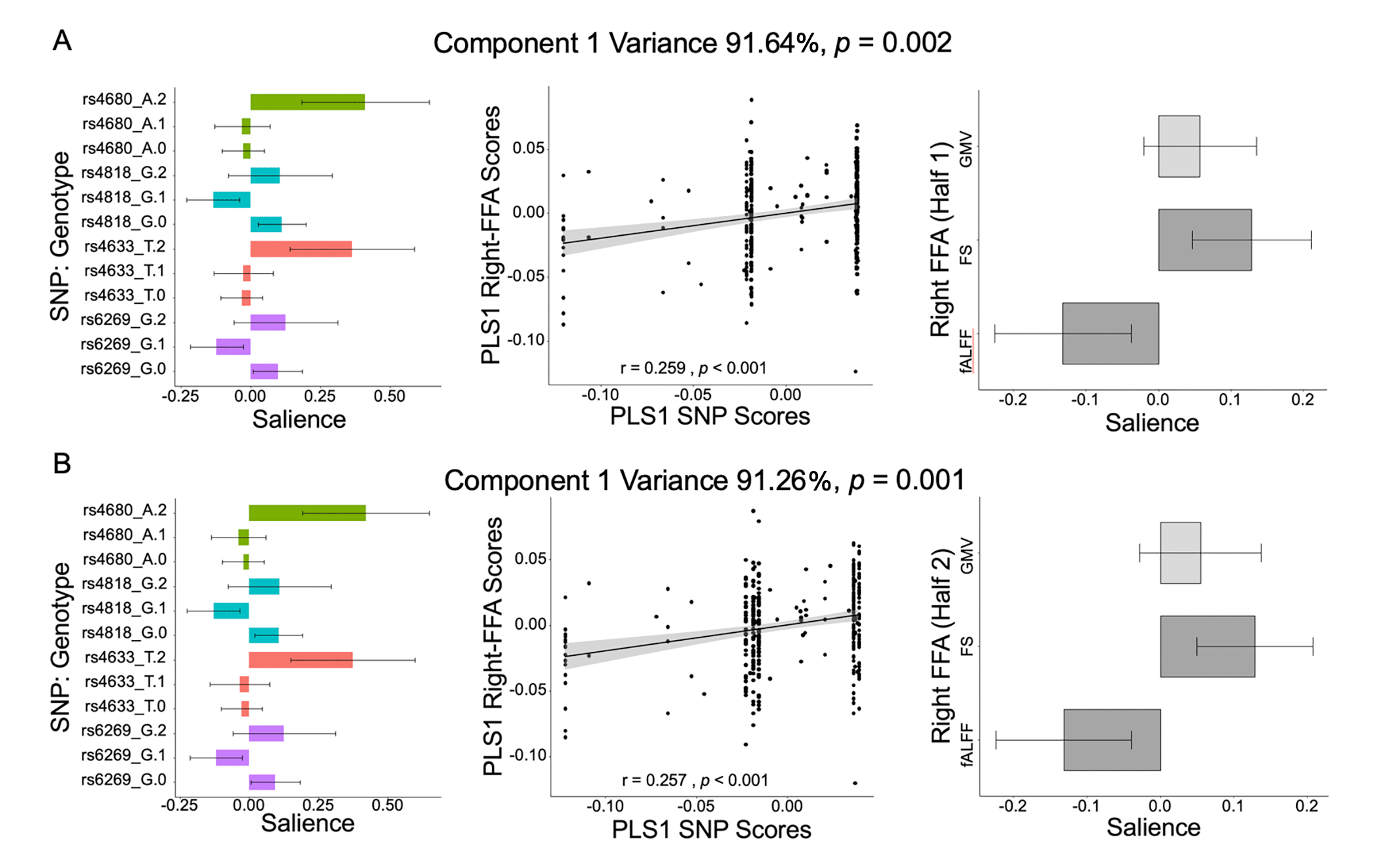


**Figure S1.** We randomly split the voxels within the rFFA into two halves and entered each half of the rFFA measures along with COMT polymorphisms into a PLSCA. (A) The PLS association between the MRI measures of the first half rFFA voxels and the COMT polymorphisms. (B) The PLS association between the MRI measures of the second half rFFA voxels and the COMT polymorphisms. The first partial least squares component (PLS1) was significant for both of the rFFA_half_-COMT associations. Left: weighted saliences for each of the COMT genotypes; middle: correlation between the first pair of latent variables (LVs). right: weighted saliences for each modal of the FFA.

**Figure S2**

**
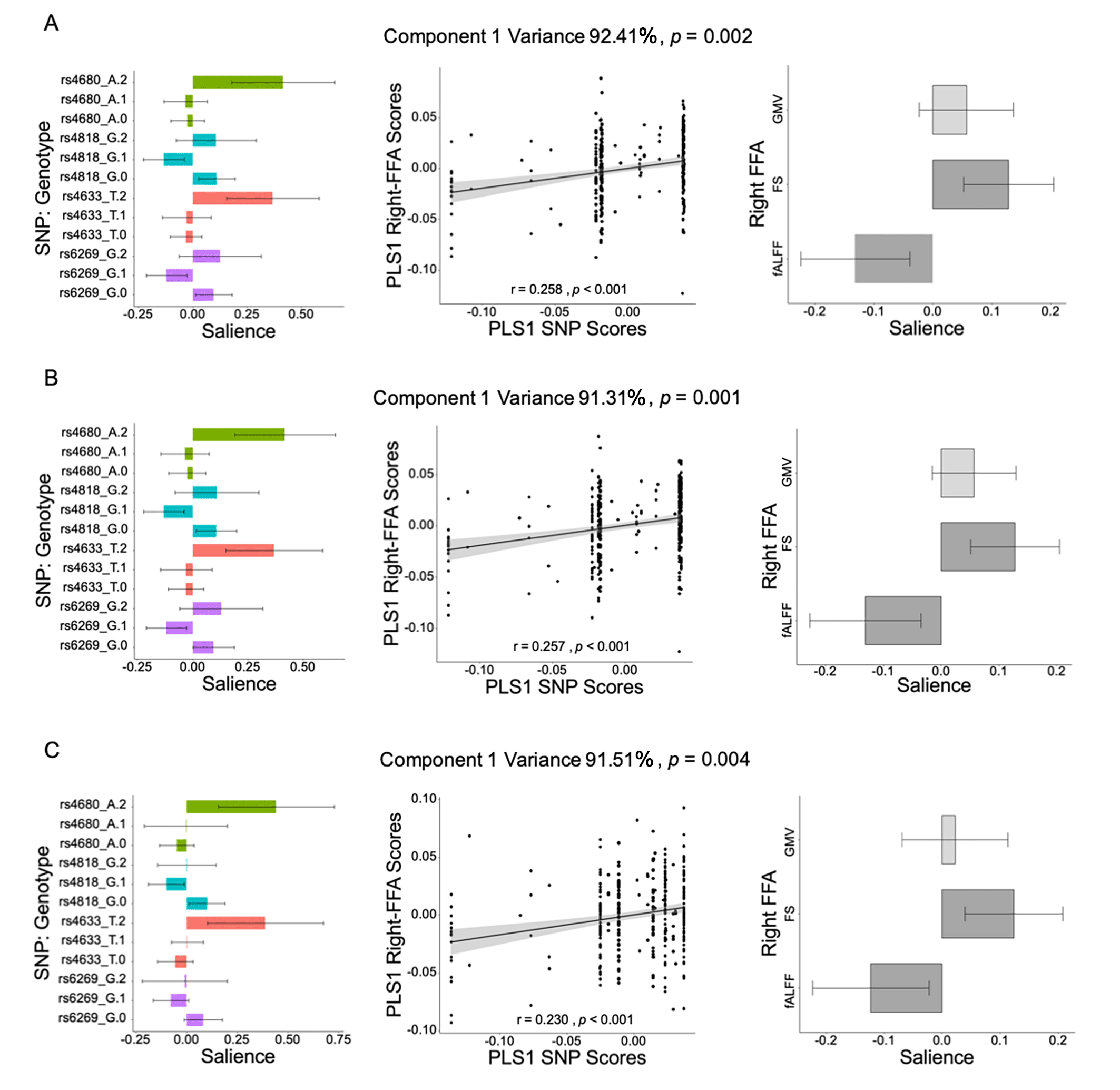
**

**Figure S2.** The PLS association between the COMT polymorphisms and the rFFA-MRI phenotypes was significant when the threshold for identifying the rFFA was (A) z = 2.71(one-tailed uncorrected p < 0.003 [FDR corrected p = 0.05]), (B) z = 2.34 (one-tailed uncorrected p < 0.01), and (C) z = 1.96 (one-tailed uncorrected p < 0.05).

**Figure S3.**


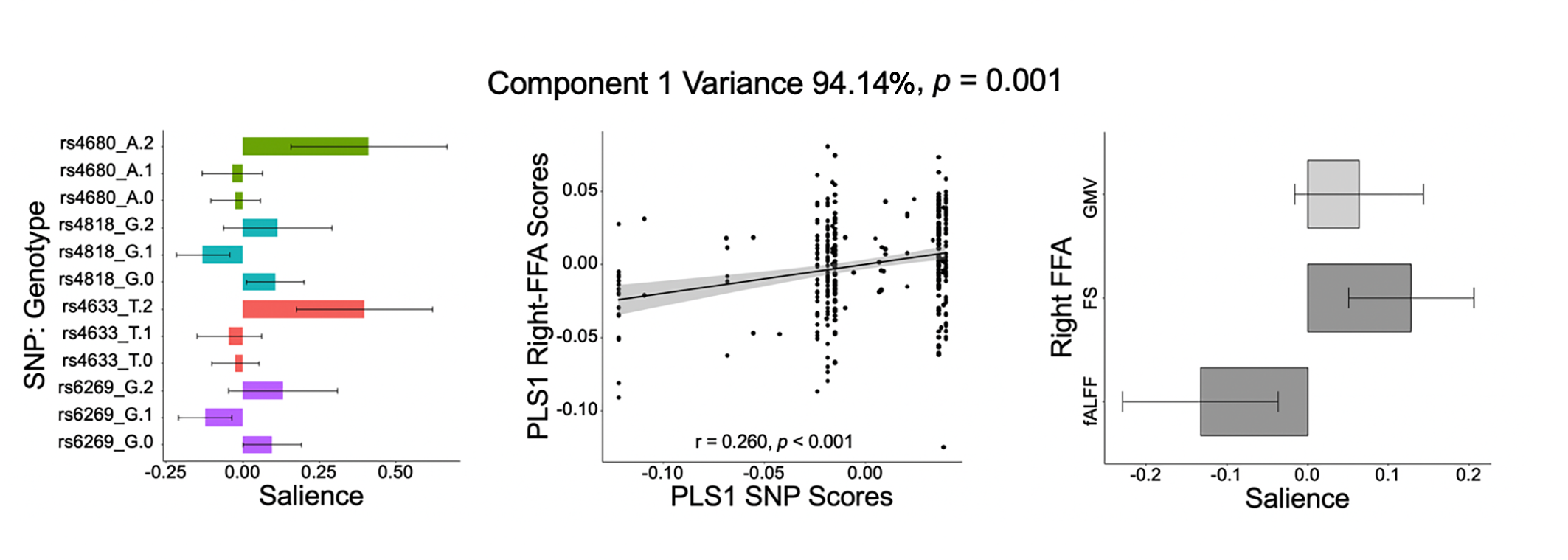


**Figure S3.** The significance of the PLS association between the COMT polymorphisms and the rFFA-MRI phenotypes still held when the total brain volume was regressed out of the GMV of the rFFA. The threshold for identifying the rFFA ROI was 2.58 (one-tailed uncorrected p < 0.005).

**Figure S4.**


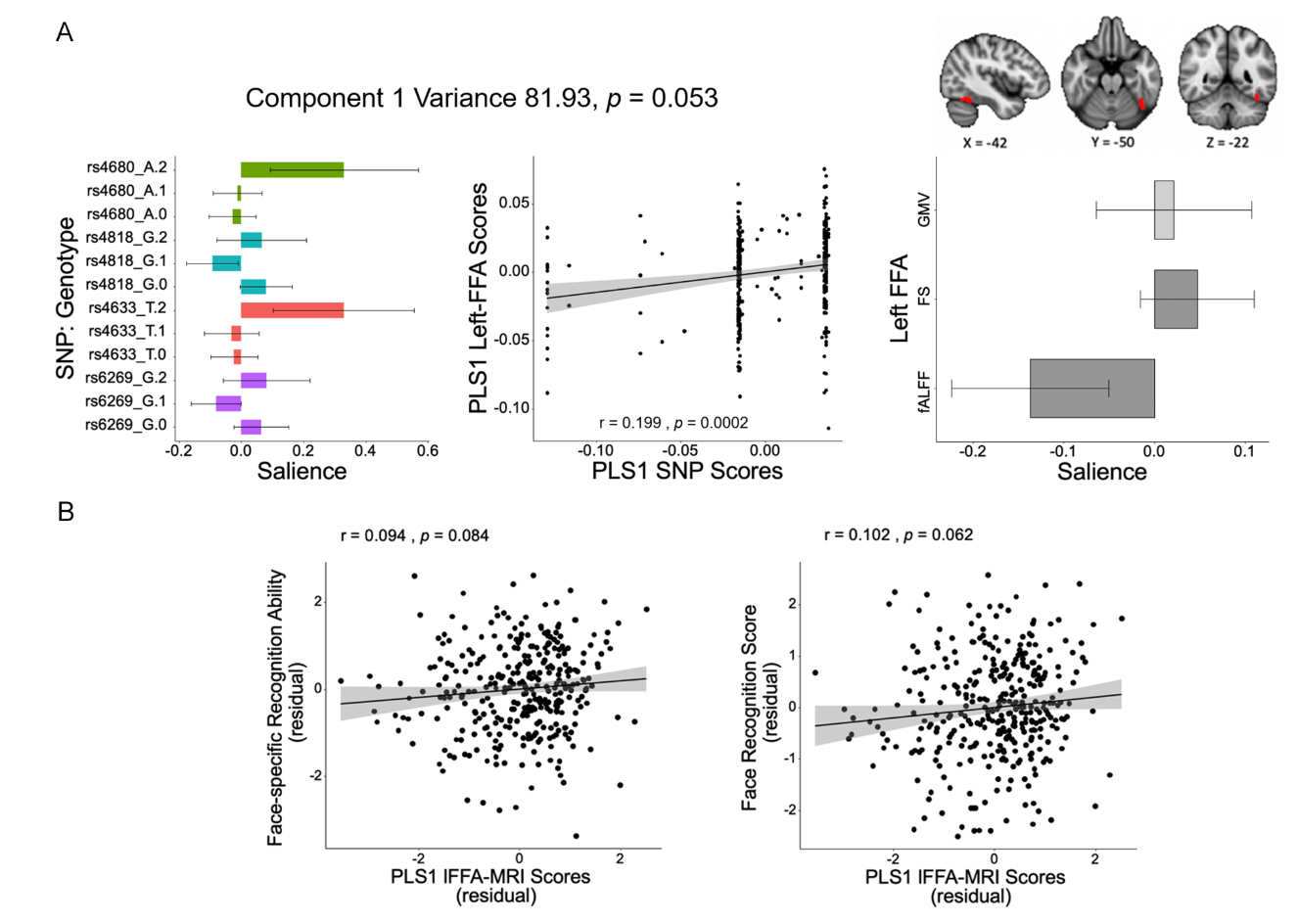


**Figure S4.** PLS association between left-FFA-MRI (lFFA-MRI) phenotypes and COMT polymorphisms. (A) The first pair of latent variables (LVs) from the PLS correlation. Left: weighted saliences for each of the COMT genotypes; right: weighted saliences (weights) for each of the MRI measures of the left FFA. (B) Associations of the PLS1 lFFA MRI scores with the face-specific recognition ability and face recognition scores.

**Figure S5.**


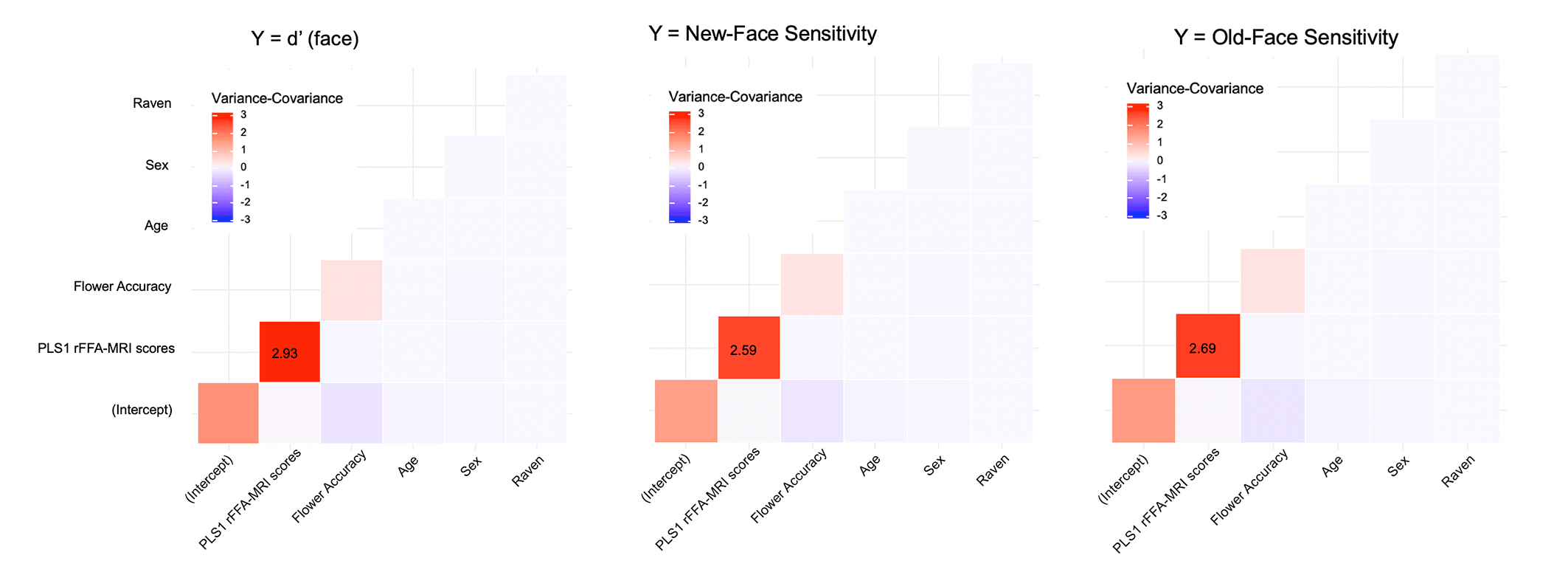


**Figure S5.** Variance-covariance matrix of the main parameters of fitted models (A) (face recognition ~ PLS1-rFFA-MRI + flower recognition + sex + age + Raven); (B) (new-face sensitivity ~ PLS1-rFFA-MRI + flower recognition + sex + age + Raven); (B) (old-face sensitivity ~ PLS1-rFFA-MRI + flower recognition + sex + age + Raven).

**Figure S6.**


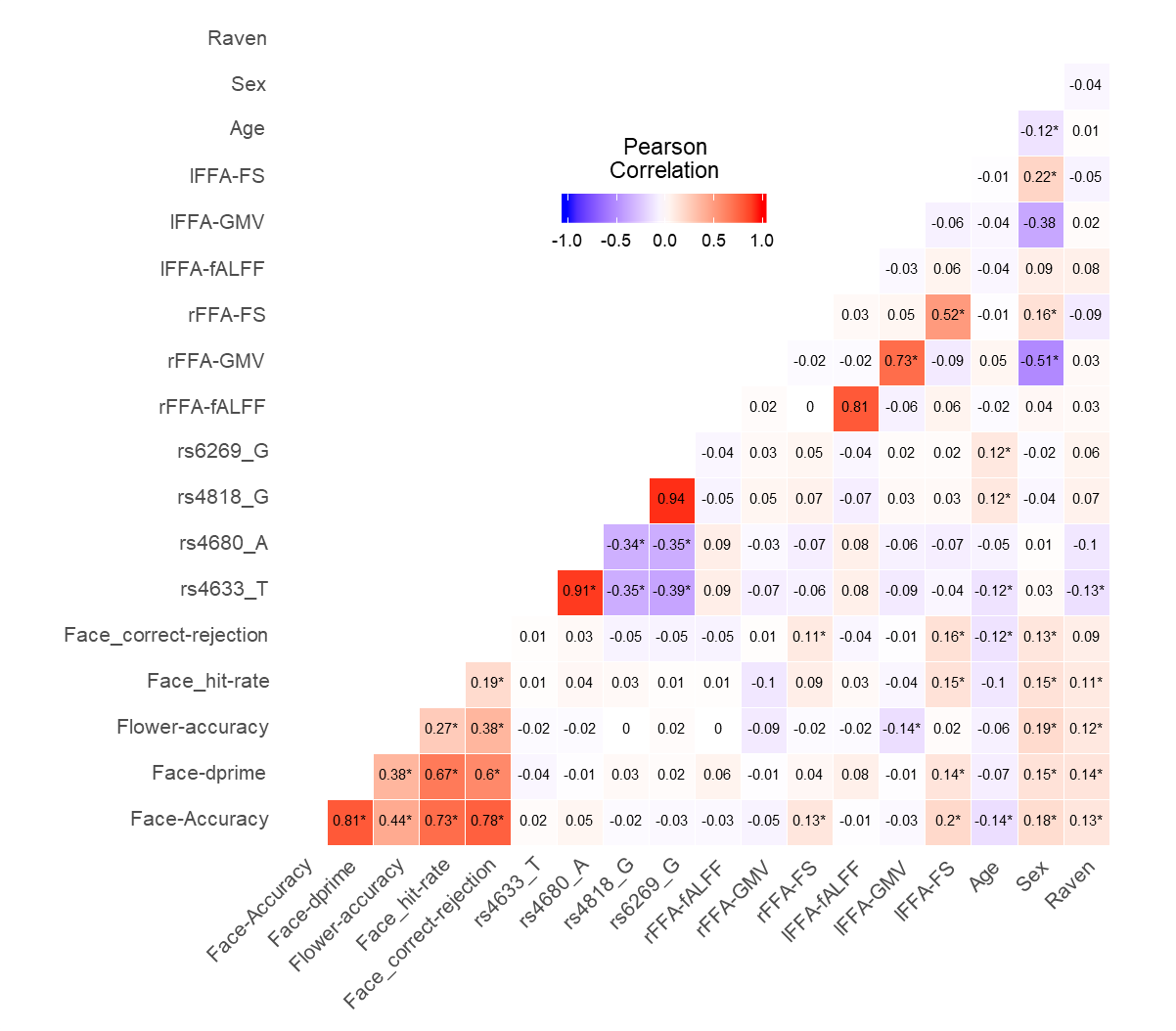


**Figure S6.** Pearson correlation matrix (lower triangular matrix) of all raw variables measured in this study (values on the diagnosis were removed).

**Table S1. Sixty-Four Cognitive-Function-Related SNPs^*^ and their Proxy Genes**

| Chr | SNP | Proxy Gene |  | Gene Function | References |
| --- | --- | --- | --- | --- | --- |
| 1 | rs1320490 | *CDC42BPA* |  | Cognitive aging | *Harris et al., 2017* |
| 2 | rs11691504 | *Intergenic* |  | General cognitive ability | *Butcher et al., 2008* |
| 2 | rs11894053 | *Intergenic* |  | Social autistic-like traits | *Ronald et al., 2010* |
| 2 | rs12613365 | *MFSD6* |  | Autisim | *Maestrini et al., 2010* |
| 3 | rs1378810 | *DNAJC13* |  | Parkinsonism | *Gustavsson et al., 2015* |
| 3 | rs53576 | *OXTR* |  | Social behavior | *Wu et al., 2005; Bosch et al., 2005* |
| 3 | rs237887 | *OXTR* |  |  |  |
| 3 | rs2254298 | *OXTR* |  |  |  |
| 3 | rs2268490 | *OXTR* |  |  |  |
| 3 | rs2251219 | *PBRM1* |  | Mental disorder risk | *Detera-Wadleigh et al., 2011; Fryland et al., 2016* |
| 5 | rs1896731 | *Intergenic* |  | Autism | *Wang et al., 2009* |
| 5 | rs4307059 | *Intergenic* |  | Autism | *Wang et al., 2009; St Pourcain et al., 2010* |
| 5 | rs17070145 | *KIBRA* |  | Memory | *Papassotiropoulos et al., 2006; Nacmias et al., 2008* |
| 6 | rs793862 | *DCDC2* |  | Language | *Meng et al., 2005; Schumacher et al., 2006* |
| 6 | rs807701 | *DCDC2* |  |  |  |
| 6 | rs1011313 | *DTNBP1* |  | Schizophrenia | *Straub et al., 2002* |
| 6 | rs9461045 | *KIAA0319* |  | Reading disorder | Harold et al., 2006; Paracchini et al., 2008 |
| 6 | rs4504469 | *KIAA0319* |  |  |  |
| 6 | rs1842129 | *NKAIN2* |  | Alzheimer's disease | Sherva et al 2014 |
| 6 | rs11154532 | *SAMD3* |  | Mathematical ability | *Docherty et al., 2010,2011* |
| 6 | rs2496143 | *TBC1D7* |  | Intellectual disability | *Capo-Chichi et al., 2013* |
| 6 | rs2143340 | *TDP2* |  | Language | *Luciano et al., 2007); Pinel et al., 2012* |
| 7 | rs8191992 | *CHRM2* |  | Alzheimer's disease | Lai et al, 2001 |
| 7 | rs2350780 | *CHRM2* |  |  |  |
| 7 | rs2061174 | *CHRM2* |  |  |  |
| 7 | rs324650 | *CHRM2* |  |  |  |
| 7 | rs851715 | *CNTNAP2* |  | Alzheimer's disease; language | Logue et al., 2011; van Abel et al., 2012 |
| 7 | rs17236239 | *CNTNAP2* |  |  |  |
| 7 | rs10246256 | *CNTNAP2* |  |  |  |
| 7 | rs2538976 | *CNTNAP2* |  |  |  |
| 7 | rs10500171 | *CNTNAP2* |  |  |  |
| 7 | rs7794745 | *CNTNAP2* |  |  |  |
| 7 | rs6947045 | *DLD* |  | Alzheimer's disease | Mastrogiacoma et al., 1996; Brown et al., 2004, 2007 |
| 7 | rs11761076 | *Intergenic* |  | Intelligence | Butcher et al., 2008 |
| 7 | rs38841 | *MET* |  | Autism/Alzheimer’disease,  schizophrenia | Campbell et al., 2006; Rudie et al., 2012; Hamasaki et al., 2014; Burdick et al., 2010 |
| 7 | rs2300052 | *NRCAM* |  | Autism | Sakurai et al., 2006; Marui et al 2009; Chakrabarti et al, 2009 |
| 8 | rs11136000 | *CLU* |  | Visual memory; Alzheimer's disease | Naj et al., 2011; Harold et al., 2009; Lambert et al., 2009 |
| 11 | rs1800497 | *ANKK1* |  | Psychosis | Mihara et al., 2003; Reuter et al., 2005 |
| 11 | rs6265 | *BDNF* |  | Memory | Kovalchuk et al., 2002; Kunugi et al., 2001 |
| 11 | rs174455 | *FADS3* |  | Mild cognitive impairment | Schuchardt et al., 2016 |
| 11 | rs2373115 | *GAB2* |  | Alzheimer’disease | Reiman et al., 2007 |
| 11 | rs1160219 | *IGSF22* |  | Schizophrenia | Yamada et al., 2011 |
| 11 | rs4754752 | *Intergenic* |  | Not clear | Meaburn et al., 2006 |
| 11 | rs11225308 | *MMP7* |  | Depression | Bobinska et al., 2016 |
| 11 | rs3851179 | *PICALM* |  | Visual memory; Alzheimer's disease | Barral et al., 2012; Harold et al., 2009; Nai et al., 2011 |
| 12 | rs12578517 | *Intergenic* |  | Autistic-like traits | Ronald et al., 2010a |
| 12 | rs1215603 | *NUAK1* |  | Mathematical ability | Docherty et al., 2010, 2011 |
| 12 | rs10505938 | *SOX5* |  | Reading disability; autism | Tran et al., 2014; Kwan 2012 |
| 14 | rs2192595 | *DPF3* |  | Neural development | Lessard et al., 2007 |
| 16 | rs7195954 | *Intergenic* |  | Intelligence | Butcher et al., 2008 |
| 16 | rs420259 | *PALB2* |  | Bipolar disorder, schizophrenia | Tesli et al., 2010) |
| 18 | rs2958182 | *TCF4* |  | Visual attention; schizophrenia | Chen et al., 2013; Stefansson et al., 2009 |
| 19 | rs429358 | *APOE-ϵ4* |  | Visual attention; Alzheimer's disease | Corder et al., 1993; Negash et al., 2009; Rusted et al., 2013; |
| 19 | rs7412 | *APOE-ϵ4* |  |  |  |
| 19 | rs6859 | *NECTIN2* |  | Alzheimer's disease | Kamboh et al 2012; Logue et al., 2011 |
| 19 | rs157580 | *TOMM40* |  | Alzheimer's disease | Hollingworth et al., 2012; Kamboh et al.,2012 |
| 19 | rs2075650 | *TOMM40* |  |  |  |
| 20 | rs1044396 | *CHRNA4* |  | Visual attention; Alzheimer's disease | Kawamata et al., 2002; Espeseth et al., 2007; Stromer et al 2012; |
| 21 | rs363449 | *GRIK1* |  | schizophrenia | Shibata et al., 2001 |
| 21 | rs2409411 | *TIAM1* |  | Spatial memory | Tsai et al., 2009; Lai et al., 2012 |
| 22 | rs4818 | *COMT* |  | Visual working memory,  visual attention,  Psychiatric disorder | Stromer et al 2012; Berryhill et al., 2013; Gruss et al., 2016; Rotondo et al., 2002; Avramopoulos et al., 2002 |
| 22 | rs4633 | *COMT* |  |  |  |
| 22 | rs6269 | *COMT* |  |  |  |
| 22 | rs4680 | *COMT* |  |  |  |

^#^ References are attached at the end of the file.

**Table S2. Parameter Estimates, Standard Errors, and Power for the PLS analysis.**

|  | Average Parameter | Estimate SD | Average SE | **Power** | Coverage |
| --- | --- | --- | --- | --- | --- |
| rFFA~SNP | 0.258 | 0.087 | 0.159 | 0.713 | 0.009 |
| SNP=~rs6269_G.1 | -0.121 | 0.157 | 0.171 | 0.065 | 0.895 |
| SNP=~rs6269_G.0 | 0.096 | 0.138 | 0.154 | 0.049 | 0.91 |
| SNP=~rs6269_G.2 | 0.127 | 0.152 | 0.165 | 0.086 | 0.884 |
| SNP=~rs4633_T.1 | -0.03 | 0.153 | 0.168 | 0.044 | 0.933 |
| SNP=~rs4633_T.0 | -0.03 | 0.153 | 0.167 | 0.056 | 0.917 |
| SNP=~rs4633_T.2 | 0.370 | 0.547 | 0.532 | 0.353 | 0.695 |
| SNP=~rs4818_G.1 | -0.132 | 0.164 | 0.176 | 0.065 | 0.888 |
| SNP=~rs4818_G.0 | 0.110 | 0.151 | 0.159 | 0.089 | 0.884 |
| SNP=~rs4818_G.2 | 0.108 | 0.149 | 0.159 | 0.067 | 0.898 |
| SNP=~rs4680_A.1 | -0.035 | 0.141 | 0.157 | 0.033 | 0.94 |
| SNP=~rs4680_A.0 | -0.024 | 0.131 | 0.149 | 0.021 | 0.96 |
| SNP=~rs4680_A.2 | 0.417 | 0.533 | 0.519 | 0.354 | 0.732 |
| rFFA=fALFF | -0.132 | 0.593 | 0.58 | 0.348 | 0.206 |
| rFFA=~GMV | 0.056 | 0.299 | 0.326 | 0.102 | 0.687 |
| rFFA=~FS | 0.129 | 0.166 | 0.209 | 0.006 | 0.923 |

The results are based on one thousand converged replications, with five hundred bootstrap resamples each (Aguirre-Urreta and Rönkkö, 2015). The results indicate a power of 0.713 for the path between rFFA and the COMT polymorphisms.

**Table S3. Pearson Correlation Coefficients and P values of the Face Recognition Accuracy, Face-specific Recognition Ability (FRA), d’ (face), Old-Face Recognition score, New-Face Recognition score, and Flower Recognition Accuracy with bilateral PLS1 SNP Scores and PLS1 FFA-MRI Scores**

| Parameter | Face Recognition Accuracy^1^ | | FRA^2^ | | d' (face) | | Old-Face Recognition^3^ | | New-Face Recognition^4^ | | Flower Recognition Accuracy | |
| --- | --- | --- | --- | --- | --- | --- | --- | --- | --- | --- | --- | --- |
|  | r | p | r | p | r | p | r | p | r | p | r | p |
| rFFA: PLS1-SNP scores | -0.005 | 0.929 | -0.004 | 0.946 | -0.022 | 0.685 | 0.027 | 0.621 | -0.036 | 0.507 | 0.004 | 0.948 |
| rFFA: PLS1-MRI scores | 0.09 | 0.097 | .119^*^ | 0.028 | 0.017 | 0.757 | 0.059 | 0.277 | .161^**^ | 0.003 | 0.039 | 0.474 |
| lFFA: PLS1-SNP scores | -0.004 | 0.942 | -0.003 | 0.954 | -0.023 | 0.677 | 0.027 | 0.62 | -0.035 | 0.526 | 0.003 | 0.963 |
| lFFA: PLS1-MRI scores | 0.069 | 0.203 | 0.073 | 0.182 | 0.027 | 0.621 | 0.037 | 0.496 | .148^**^ | 0.006 | -0.009 | 0.868 |
| Sex | .181^**^ | 0.001 | .109^*^ | 0.045 | .151^**^ | 0.005 | .183^**^ | 0.001 | .155^**^ | 0.004 | .190^**^ | 0 |
| Age | -.145^**^ | 0.008 | -.134^*^ | 0.014 | -0.075 | 0.171 | -.131^*^ | 0.016 | -.133^*^ | 0.015 | -0.056 | 0.305 |
| Raven | .130^*^ | 0.017 | 0.088 | 0.105 | .136^*^ | 0.012 | 0.103 | 0.059 | .145^**^ | 0.008 | .116^*^ | 0.033 |
| Flower Recognition Accuracy | .436^***^ | < 0.001 | 0 | 1 | .377^***^ | < 0.001 | .399^***^ | < 0.001 | 0.390^***^ | < 0.001 | 1 | 0 |

^1^ Face recognition accuracy was calculated as the face recognition accuracy (the average proportion of hits and correct rejections) for faces, and flower recognition accuracy was calculated as the flower recognition accuracy (the average proportion of hits and correct rejections) for flowers. ^2^ FRA was calculated as the normalized residual of the face recognition accuracy score after regressing out the flower recognition accuracy score. ^3^Old-Face recognition score was calculated as the normalized residual of the old face recognition accuracy (hit rate) after regressing out the response bias, and ^4^new-face recognition score was calculated as the normalized residual of the new face recognition accuracy (correct rejection rate) after regressing out the response bias.

^***^ uncorrected p < 0.001 (2-tailed). ^**^ uncorrected p < 0.01 (2-tailed). ^*^ uncorrected p < 0.05 (2-tailed).

**References for Table 1**

Almeida OP, Schwab SG, Lautenschlager NT, et al. (2008) KIBRA genetic polymorphism influences episodic memory in later life, but does not increase the risk of mild cognitive impairment. J Cell Mol Med 12: 1672-1676.

Avramopoulos D, Stefanis NC, Hantoumi I, et al. (2002) Higher scores of self reported schizotypy in healthy young males carrying the COMT high activity allele. Mol Psychiatry 7: 706-711.

Bartko G, Herczeg I and Zador G. (1988) Clinical symptomatology and drug compliance in schizophrenic patients. Acta Psychiatr Scand 77: 74-76.

Bates TC, Price JF, Harris SE, et al. (2009) Association of KIBRA and memory. Neurosci Lett 458: 140-143.

Berryhill ME, Wiener M, Stephens JA, et al. (2013) COMT and ANKK1-Taq-Ia genetic polymorphisms influence visual working memory. PLoS One 8: e55862.

Bobinska K, Szemraj J, Czarny P, et al. (2016) Expression and Activity of Metalloproteinases in Depression. Med Sci Monit 22: 1334-1341.

Bosch OJ, Meddle SL, Beiderbeck DI, et al. (2005) Brain oxytocin correlates with maternal aggression: link to anxiety. J Neurosci 25: 6807-6815.

Brown AM, Gordon D, Lee H, et al. (2004) Association of the dihydrolipoamide dehydrogenase gene with Alzheimer's disease in an Ashkenazi Jewish population. Am J Med Genet B Neuropsychiatr Genet 131b: 60-66.

Brown AM, Gordon D, Lee H, et al. (2007) Testing for linkage and association across the dihydrolipoyl dehydrogenase gene region with Alzheimer's disease in three sample populations. Neurochem Res 32: 857-869.

Butcher LM, Davis OS, Craig IW, et al. (2008) Genome-wide quantitative trait locus association scan of general cognitive ability using pooled DNA and 500K single nucleotide polymorphism microarrays. Genes Brain Behav 7: 435-446.

Campbell DB, Sutcliffe JS, Ebert PJ, et al. (2006) A genetic variant that disrupts MET transcription is associated with autism. Proc Natl Acad Sci U S A 103: 16834-16839.

Capo-Chichi JM, Tcherkezian J, Hamdan FF, et al. (2013) Disruption of TBC1D7, a subunit of the TSC1-TSC2 protein complex, in intellectual disability and megalencephaly. J Med Genet 50: 740-744.

Chakrabarti B, Dudbridge F, Kent L, et al. (2009) Genes related to sex steroids, neural growth, and social&ndash;emotional behavior are associated with autistic traits, empathy, and Asperger syndrome. Autism Research 2: 157-177.

Corder EH, Saunders AM, Strittmatter WJ, et al. (1993) Gene dose of apolipoprotein E type 4 allele and the risk of Alzheimer's disease in late onset families. Science 261: 921-923.

Detera-Wadleigh SD and Akula N. (2011) A systems approach to the biology of mood disorders through network analysis of candidate genes. Pharmacopsychiatry 44 Suppl 1: S35-42.

Docherty SJ, Kovas Y, Petrill SA, et al. (2010) Generalist genes analysis of DNA markers associated with mathematical ability and disability reveals shared influence across ages and abilities. BMC Genet 11: 61.

Docherty SJ, Kovas Y and Plomin R. (2011) Gene-environment interaction in the etiology of mathematical ability using SNP sets. Behav Genet 41: 141-154.

Espeseth T, Endestad T, Rootwelt H, et al. (2007) Nicotine receptor gene CHRNA4 modulates early event-related potentials in auditory and visual oddball target detection tasks. Neuroscience 147: 974-985.

Fryland T, Christensen JH, Pallesen J, et al. (2016) Identification of the BRD1 interaction network and its impact on mental disorder risk. Genome Med 8: 53.

Gruss LF, Langaee T and Keil A. (2016) The role of the COMT val158met polymorphism in mediating aversive learning in visual cortex. Neuroimage 125: 633-642.

Gustavsson EK, Trinh J, Guella I, et al. (2015) DNAJC13 genetic variants in parkinsonism. Mov Disord 30: 273-278.

Hamasaki H, Honda H, Suzuki SO, et al. (2014) Down-regulation of MET in hippocampal neurons of Alzheimer's disease brains. Neuropathology 34: 284-290.

Harold D, Abraham R, Hollingworth P, et al. (2009) Genome-wide association study identifies variants at CLU and PICALM associated with Alzheimer's disease. Nat Genet 41: 1088-1093.

Harold D, Paracchini S, Scerri T, et al. (2006) Further evidence that the KIAA0319 gene confers susceptibility to developmental dyslexia. Mol Psychiatry 11: 1085-1091, 1061.

Harris SE, Riggio V, Evenden L, et al. (2017) Age-related gene expression changes, and transcriptome wide association study of physical and cognitive aging traits, in the Lothian Birth Cohort 1936. Aging (Albany NY) 9: 2489-2503.

Hollingworth P, Sweet R, Sims R, et al. (2012) Genome-wide association study of Alzheimer's disease with psychotic symptoms. Mol Psychiatry 17: 1316-1327.

Kamboh MI, Barmada MM, Demirci FY, et al. (2012) Genome-wide association analysis of age-at-onset in Alzheimer's disease. Mol Psychiatry 17: 1340-1346.

Kovalchuk Y, Hanse E, Kafitz KW, et al. (2002) Postsynaptic Induction of BDNF-Mediated Long-Term Potentiation. Science 295: 1729-1734.

Kunugi H, Ueki A, Otsuka M, et al. (2001) A novel polymorphism of the brain-derived neurotrophic factor (BDNF) gene associated with late-onset Alzheimer's disease. Mol Psychiatry 6: 83-86.

Kwan KY. (2013) Transcriptional dysregulation of neocortical circuit assembly in ASD. Int Rev Neurobiol 113: 167-205.

Lai KO, Wong AS, Cheung MC, et al. (2012) TrkB phosphorylation by Cdk5 is required for activity-dependent structural plasticity and spatial memory. Nat Neurosci 15: 1506-1515.

Lai MK, Lai OF, Keene J, et al. (2001) Psychosis of Alzheimer's disease is associated with elevated muscarinic M2 binding in the cortex. Neurology 57: 805-811.

Lessard J, Wu JI, Ranish JA, et al. (2007) An essential switch in subunit composition of a chromatin remodeling complex during neural development. Neuron 55: 201-215.

Logue MW, Schu M, Vardarajan BN, et al. (2011) A comprehensive genetic association study of Alzheimer disease in African Americans. Arch Neurol 68: 1569-1579.

Luciano M, Lind PA, Duffy DL, et al. (2007) A haplotype spanning KIAA0319 and TTRAP is associated with normal variation in reading and spelling ability. Biol Psychiatry 62: 811-817.

Maestrini E, Pagnamenta AT, Lamb JA, et al. (2010) High-density SNP association study and copy number variation analysis of the AUTS1 and AUTS5 loci implicate the IMMP2L-DOCK4 gene region in autism susceptibility. Mol Psychiatry 15: 954-968.

Marui T, Funatogawa I, Koishi S, et al. (2009) Association of the neuronal cell adhesion molecule (NRCAM) gene variants with autism. Int J Neuropsychopharmacol 12: 1-10.

Mastrogiacoma F, Lindsay JG, Bettendorff L, et al. (1996) Brain protein and alpha-ketoglutarate dehydrogenase complex activity in Alzheimer's disease. Ann Neurol 39: 592-598.

Meaburn E, Butcher LM, Schalkwyk LC, et al. (2006) Genotyping pooled DNA using 100K SNP microarrays: a step towards genomewide association scans. Nucleic Acids Res 34: e27.

Meng H, Smith SD, Hager K, et al. (2005) DCDC2 is associated with reading disability and modulates neuronal development in the brain. Proc Natl Acad Sci U S A 102: 17053-17058.

Mihara K, Kondo T, Suzuki A, et al. (2003) Relationship between functional dopamine D2 and D3 receptors gene polymorphisms and neuroleptic malignant syndrome. Am J Med Genet B Neuropsychiatr Genet 117b: 57-60.

Nacmias B, Bessi V, Bagnoli S, et al. (2008) KIBRA gene variants are associated with episodic memory performance in subjective memory complaints. Neurosci Lett 436: 145-147.

Naj AC, Jun G, Beecham GW, et al. (2011) Common variants at MS4A4/MS4A6E, CD2AP, CD33 and EPHA1 are associated with late-onset Alzheimer's disease. Nat Genet 43: 436-441.

Negash S, Greenwood PM, Sunderland T, et al. (2009) The influence of apolipoprotein E genotype on visuospatial attention dissipates after age 80. Neuropsychology 23: 81-89.

Papassotiropoulos A, Stephan DA, Huentelman MJ, et al. (2006) Common Kibra alleles are associated with human memory performance. Science 314: 475-478.

Paracchini S, Thomas A, Castro S, et al. (2006) The chromosome 6p22 haplotype associated with dyslexia reduces the expression of KIAA0319, a novel gene involved in neuronal migration. Hum Mol Genet 15: 1659-1666.

Pinel P, Fauchereau F, Moreno A, et al. (2012) Genetic variants of FOXP2 and KIAA0319/TTRAP/THEM2 locus are associated with altered brain activation in distinct language-related regions. J Neurosci 32: 817-825.

Reiman EM, Webster JA, Myers AJ, et al. (2007) GAB2 alleles modify Alzheimer's risk in APOE epsilon4 carriers. Neuron 54: 713-720.

Reuter M, Peters K, Schroeter K, et al. (2005) The influence of the dopaminergic system on cognitive functioning: A molecular genetic approach. Behav Brain Res 164: 93-99.

Ronald A, Butcher LM, Docherty S, et al. (2010a) A genome-wide association study of social and non-social autistic-like traits in the general population using pooled DNA, 500 K SNP microarrays and both community and diagnosed autism replication samples. Behav Genet 40: 31-45.

Ronald A, Butcher LM, Docherty S, et al. (2010b) A genome-wide association study of social and non-social autistic-like traits in the general population using pooled DNA, 500 K SNP microarrays and both community and diagnosed autism replication samples. Behav Genet 40: 31-45.

Rotondo A, Mazzanti C, Dell'Osso L, et al. (2002) Catechol o-methyltransferase, serotonin transporter, and tryptophan hydroxylase gene polymorphisms in bipolar disorder patients with and without comorbid panic disorder. Am J Psychiatry 159: 23-29.

Rudie JD, Hernandez LM, Brown JA, et al. (2012) Autism-associated promoter variant in MET impacts functional and structural brain networks. Neuron 75: 904-915.

Rusted JM, Evans SL, King SL, et al. (2013) APOE e4 polymorphism in young adults is associated with improved attention and indexed by distinct neural signatures. Neuroimage 65: 364-373.

Schuchardt JP, Kobe T, Witte V, et al. (2016) Genetic Variants of the FADS Gene Cluster Are Associated with Erythrocyte Membrane LC PUFA Levels in Patients with Mild Cognitive Impairment. J Nutr Health Aging 20: 611-620.

Schumacher J, Anthoni H, Dahdouh F, et al. (2006) Strong genetic evidence of DCDC2 as a susceptibility gene for dyslexia. Am J Hum Genet 78: 52-62.

Sherva R, Tripodis Y, Bennett DA, et al. (2014) Genome-wide association study of the rate of cognitive decline in Alzheimer's disease. Alzheimers Dement 10: 45-52.

Shibata H, Joo A, Fujii Y, et al. (2001) Association study of polymorphisms in the GluR5 kainate receptor gene (GRIK1) with schizophrenia. Psychiatr Genet 11: 139-144.

St Pourcain B, Wang K, Glessner JT, et al. (2010) Association between a high-risk autism locus on 5p14 and social communication spectrum phenotypes in the general population. Am J Psychiatry 167: 1364-1372.

Stefansson H, Ophoff RA, Steinberg S, et al. (2009) Common variants conferring risk of schizophrenia. Nature 460: 744-747.

Stormer VS, Passow S, Biesenack J, et al. (2012) Dopaminergic and cholinergic modulations of visual-spatial attention and working memory: insights from molecular genetic research and implications for adult cognitive development. Dev Psychol 48: 875-889.

Straub RE, Jiang Y, MacLean CJ, et al. (2002) Genetic variation in the 6p22.3 gene DTNBP1, the human ortholog of the mouse dysbindin gene, is associated with schizophrenia. Am J Hum Genet 71: 337-348.

Tesli M, Athanasiu L, Mattingsdal M, et al. (2010) Association analysis of PALB2 and BRCA2 in bipolar disorder and schizophrenia in a scandinavian case-control sample. Am J Med Genet B Neuropsychiatr Genet 153b: 1276-1282.

Tran C, Wigg KG, Zhang K, et al. (2014) Association of the ROBO1 gene with reading disabilities in a family-based analysis. Genes Brain Behav 13: 430-438.

Tsai SY, Hayashi T, Harvey BK, et al. (2009) Sigma-1 receptors regulate hippocampal dendritic spine formation via a free radical-sensitive mechanism involving Rac1xGTP pathway. Proc Natl Acad Sci U S A 106: 22468-22473.

van Abel D, Michel O, Veerhuis R, et al. (2012) Direct downregulation of CNTNAP2 by STOX1A is associated with Alzheimer's disease. J Alzheimers Dis 31: 793-800.

Wang K, Zhang H, Ma D, et al. (2009) Common genetic variants on 5p14.1 associate with autism spectrum disorders. Nature 459: 528-533.

Wu S, Jia M, Ruan Y, et al. (2005) Positive association of the oxytocin receptor gene (OXTR) with autism in the Chinese Han population. Biol Psychiatry 58: 74-77.

Yamada K, Iwayama Y, Hattori E, et al. (2011) Genome-wide association study of schizophrenia in Japanese population. PLoS One 6: e20468-e20468.
